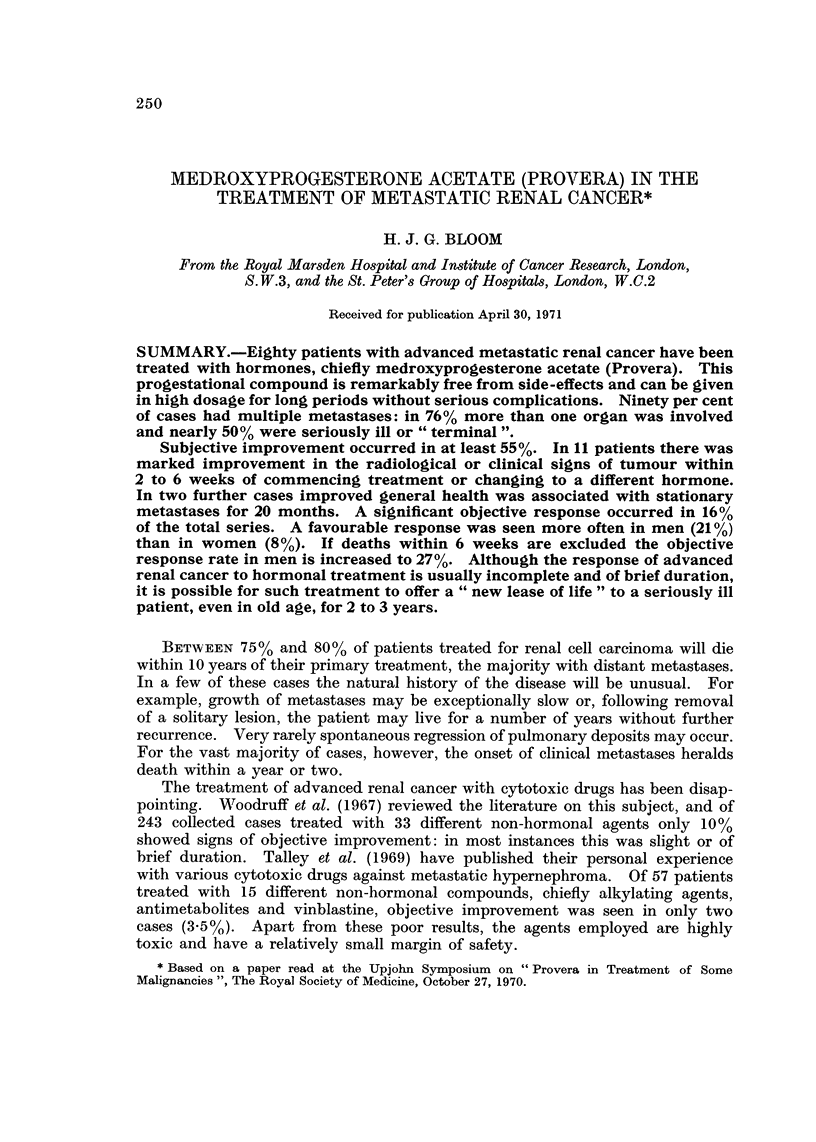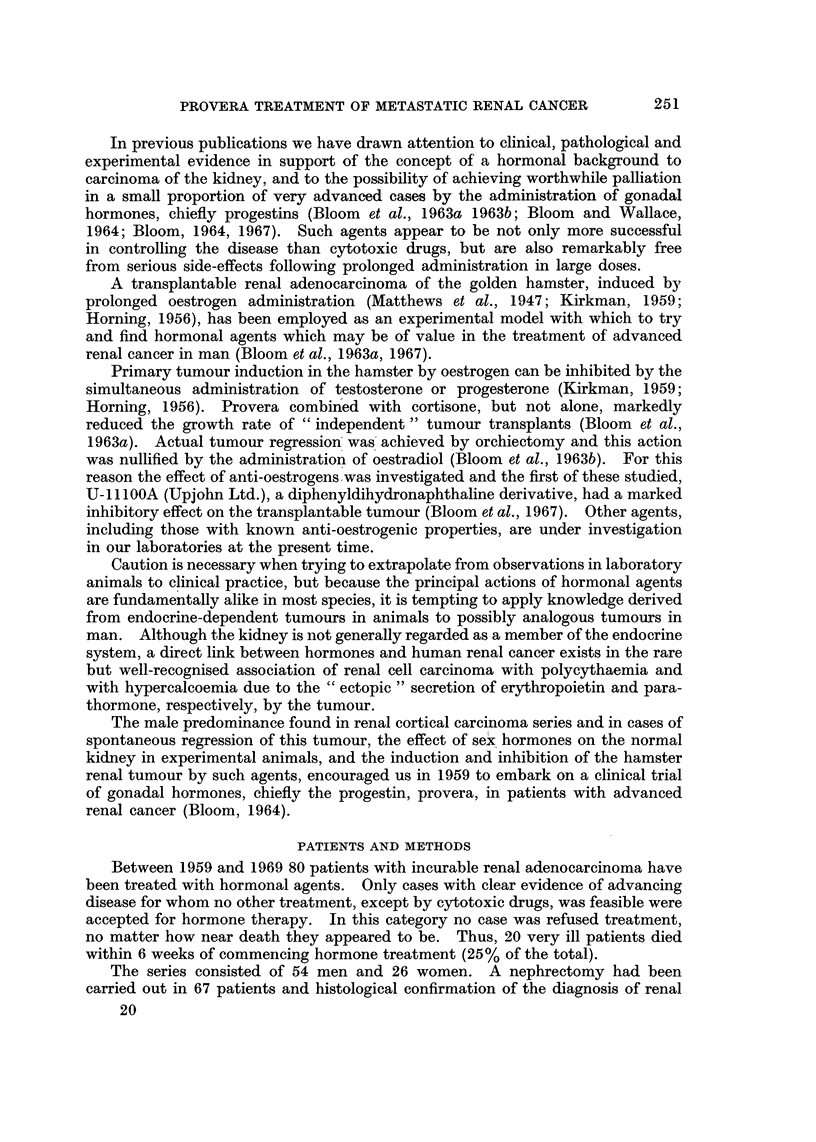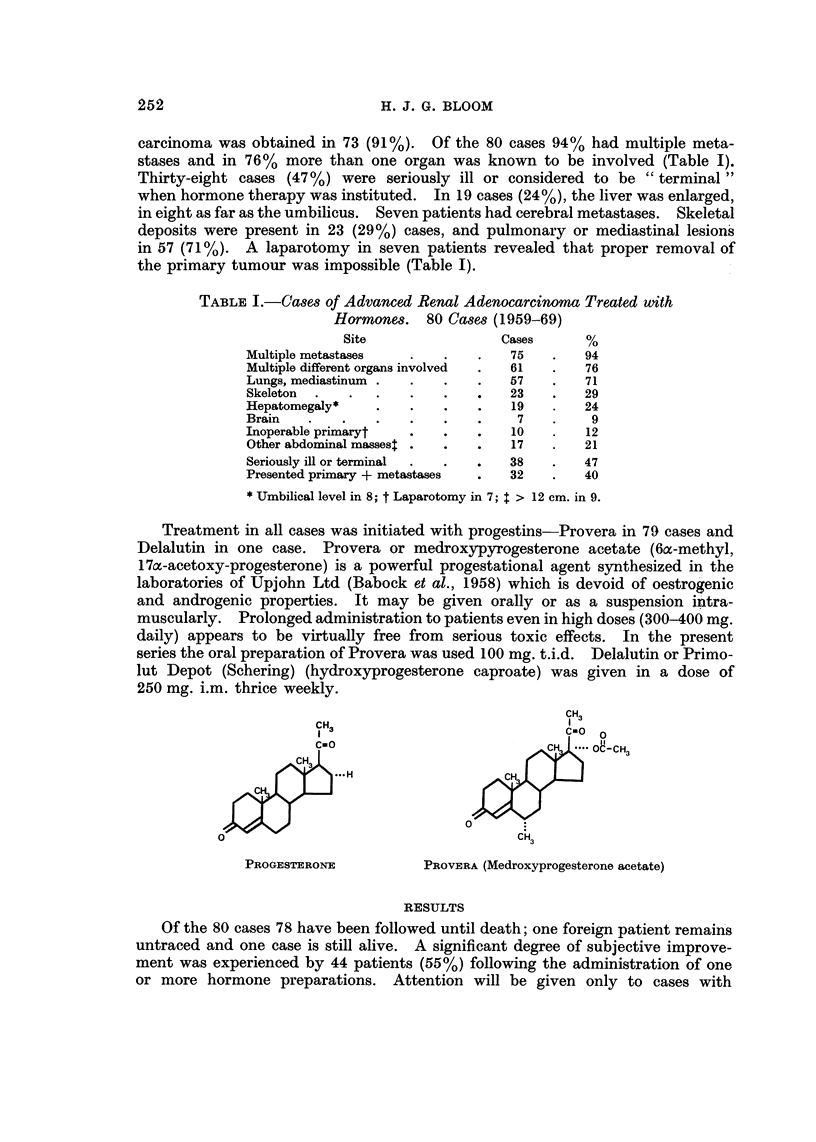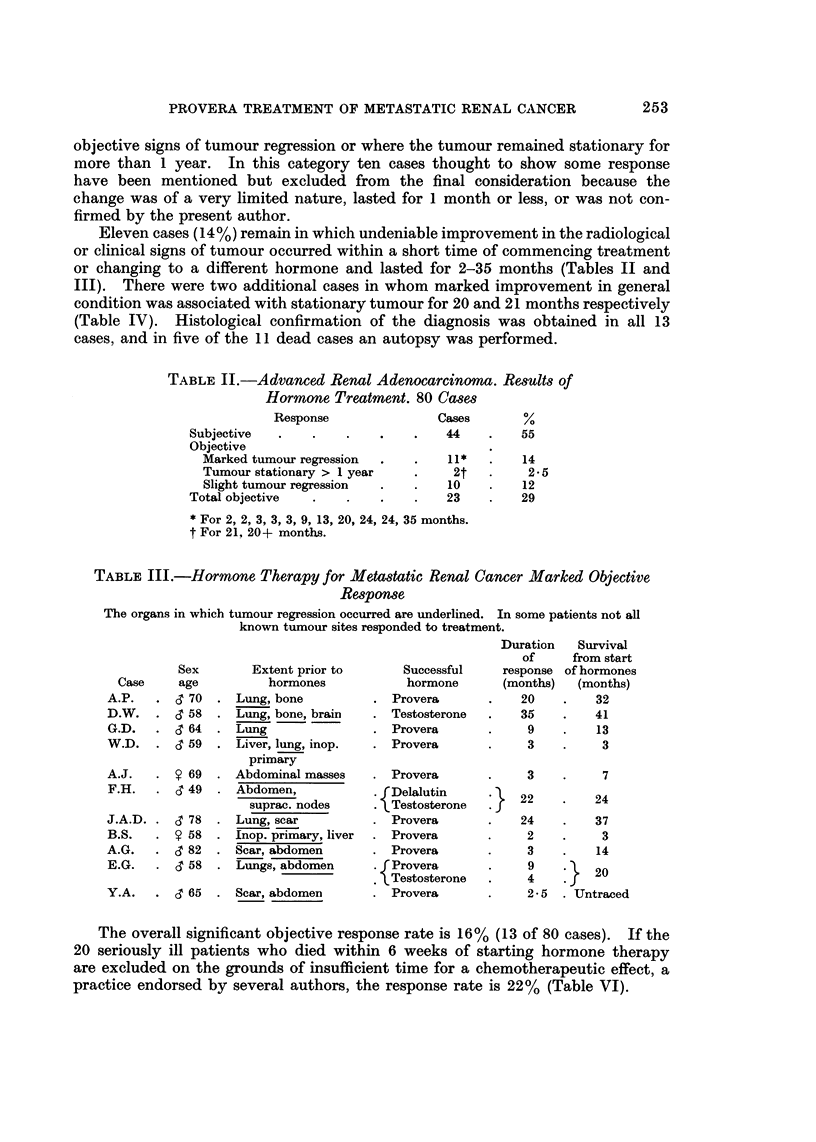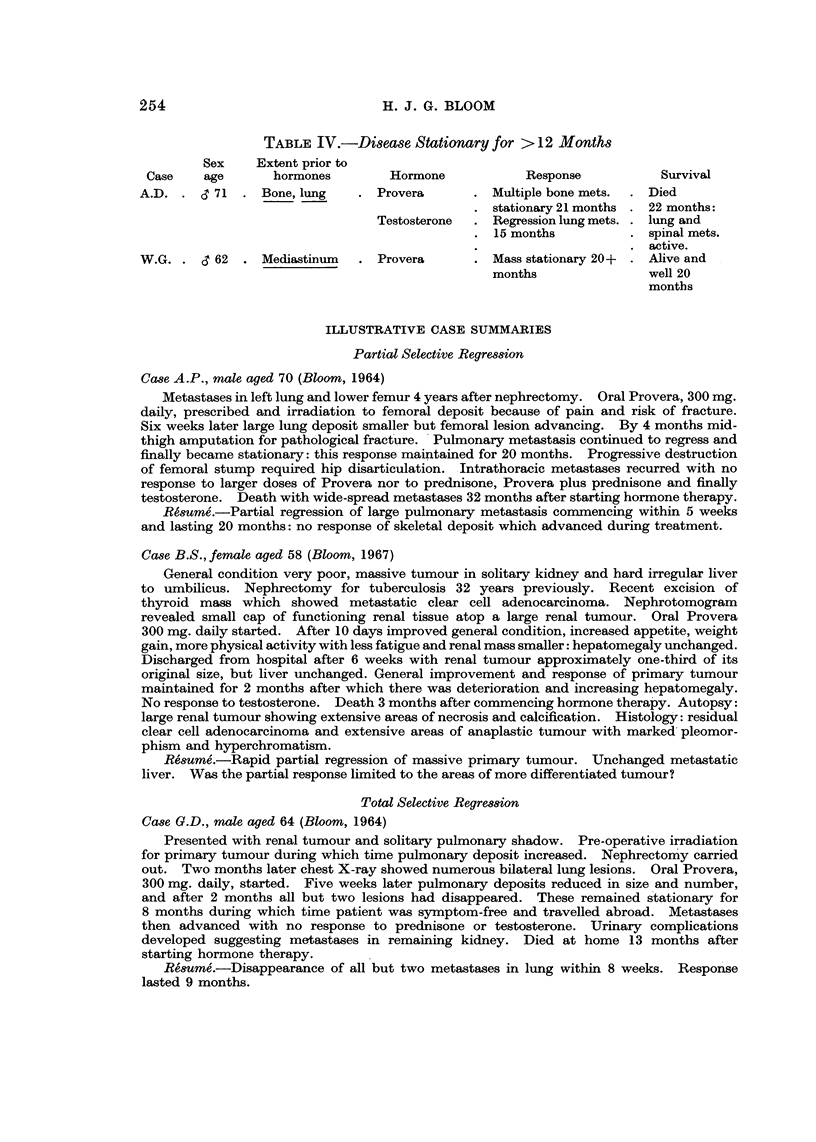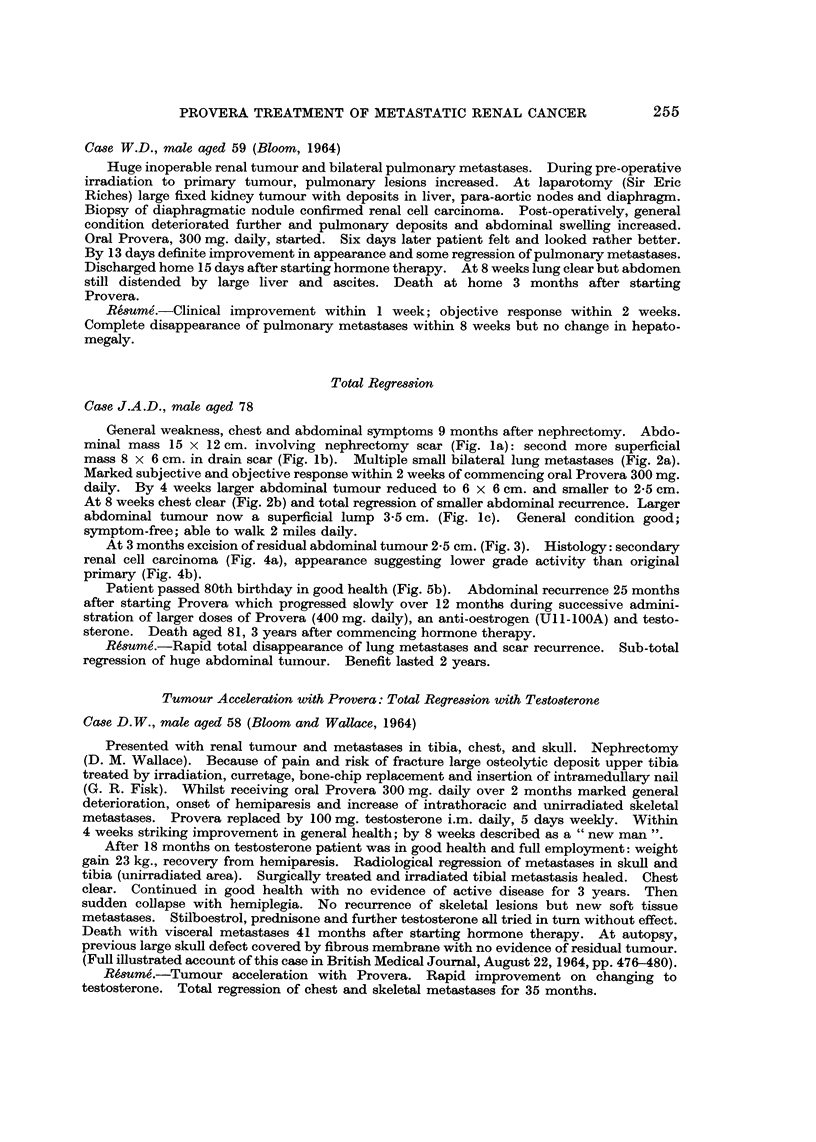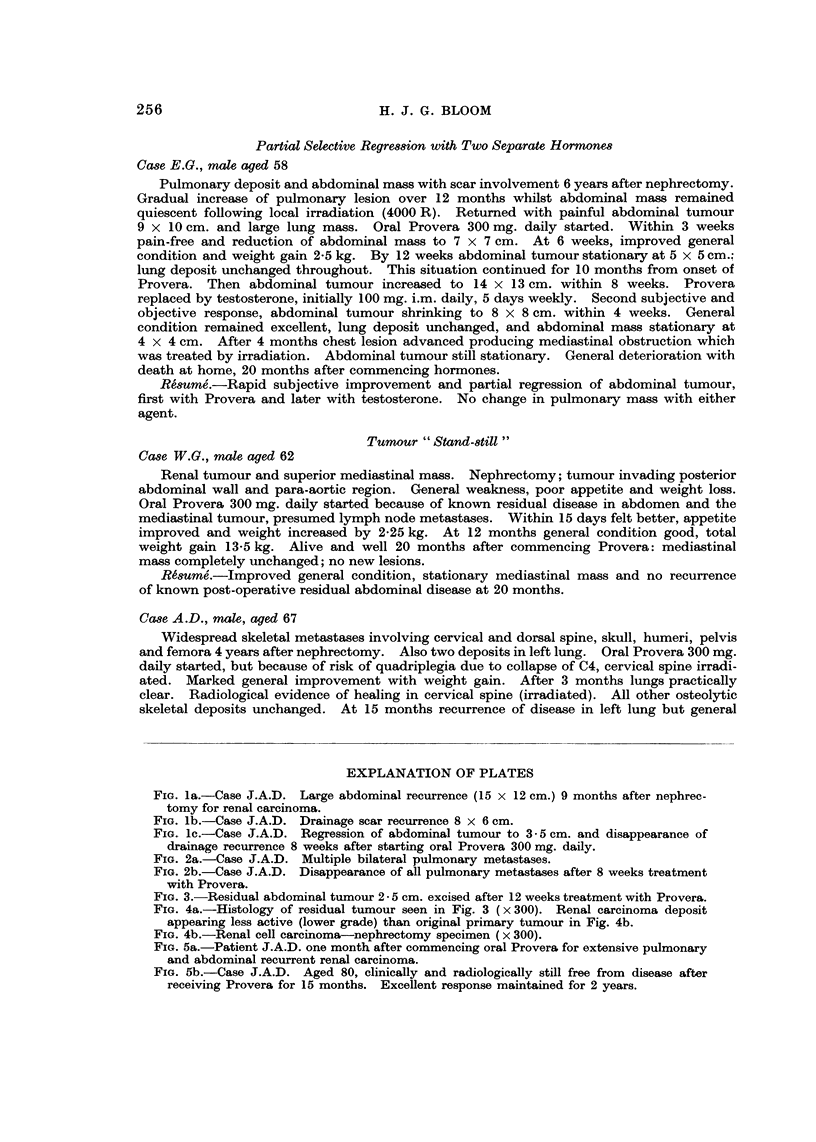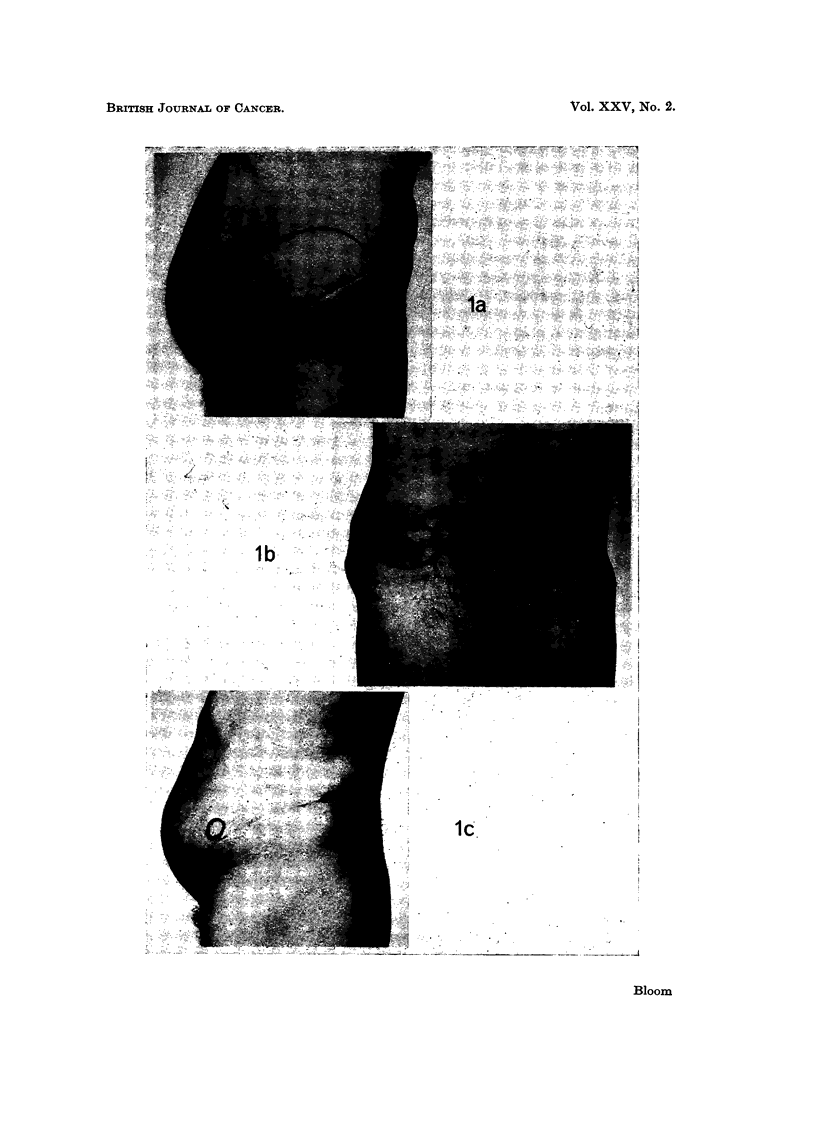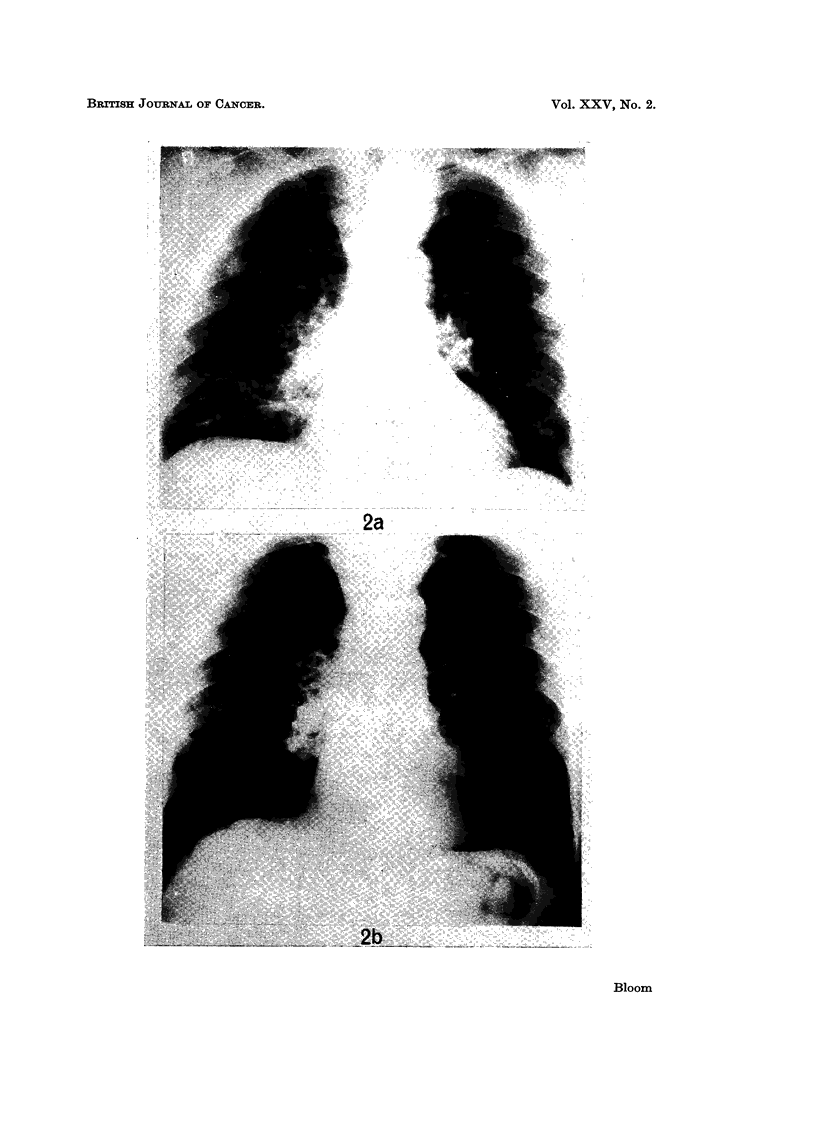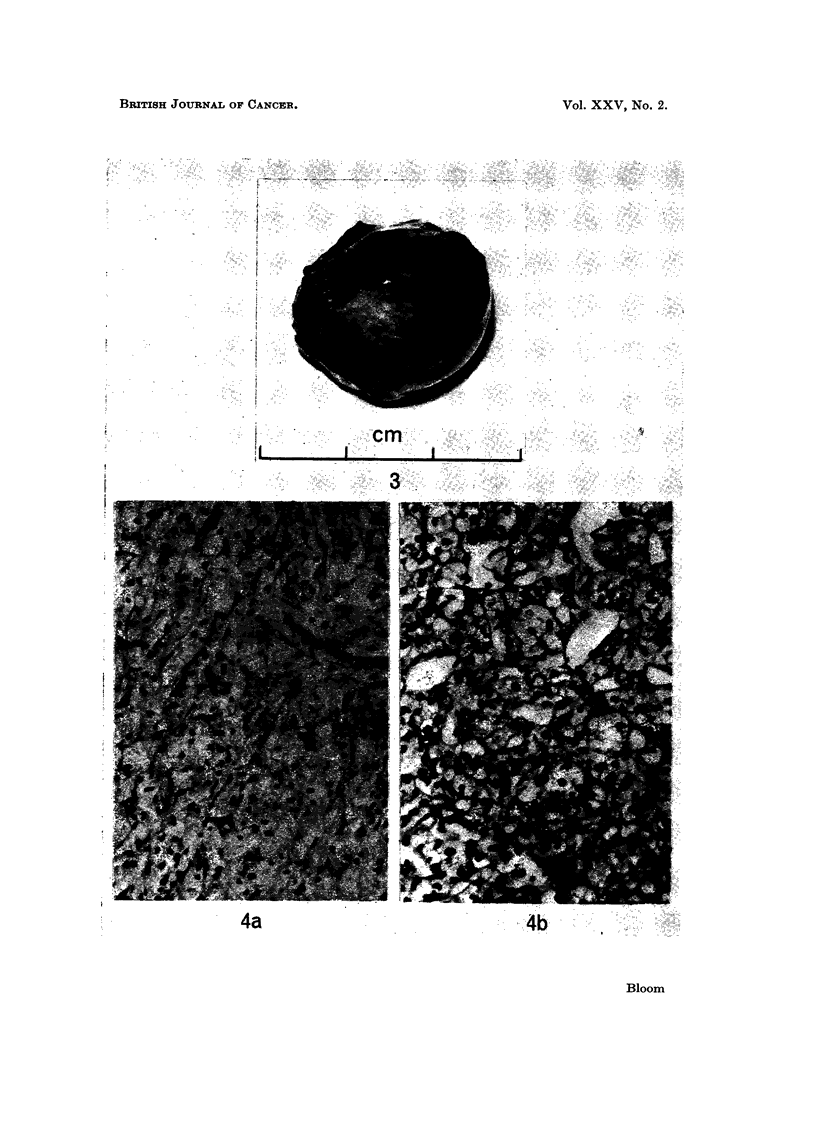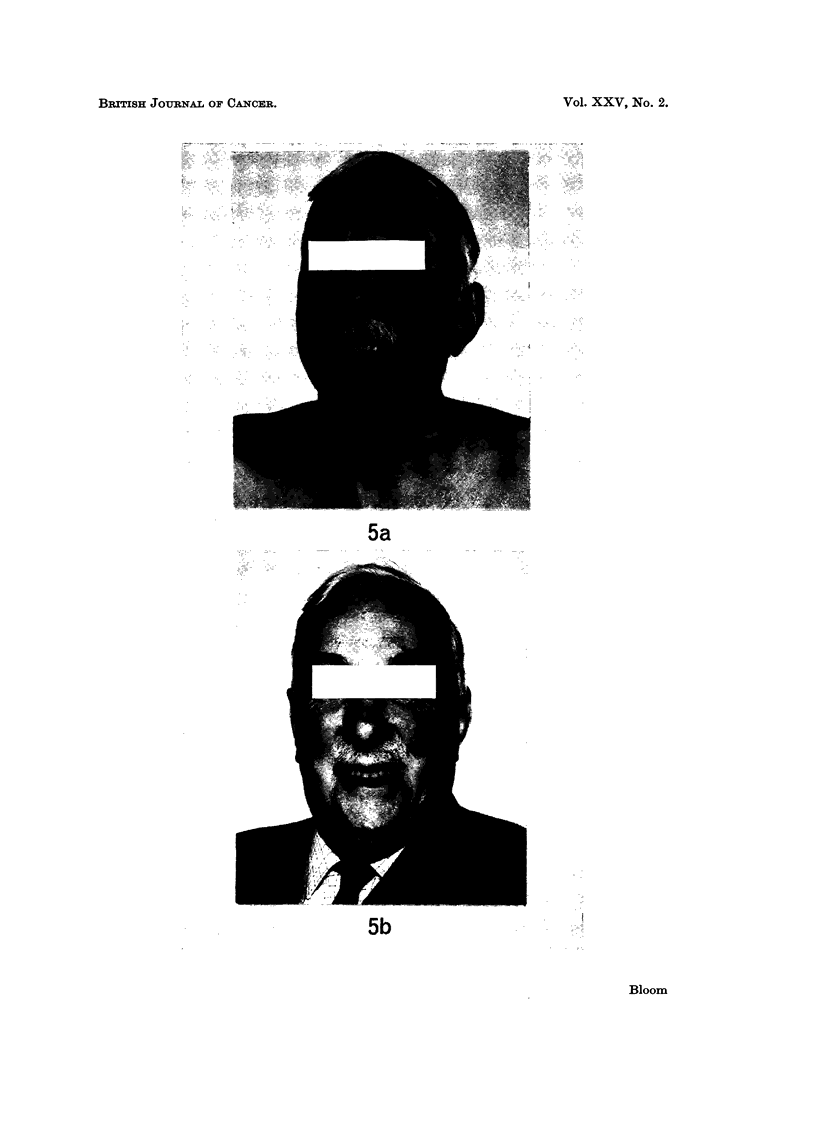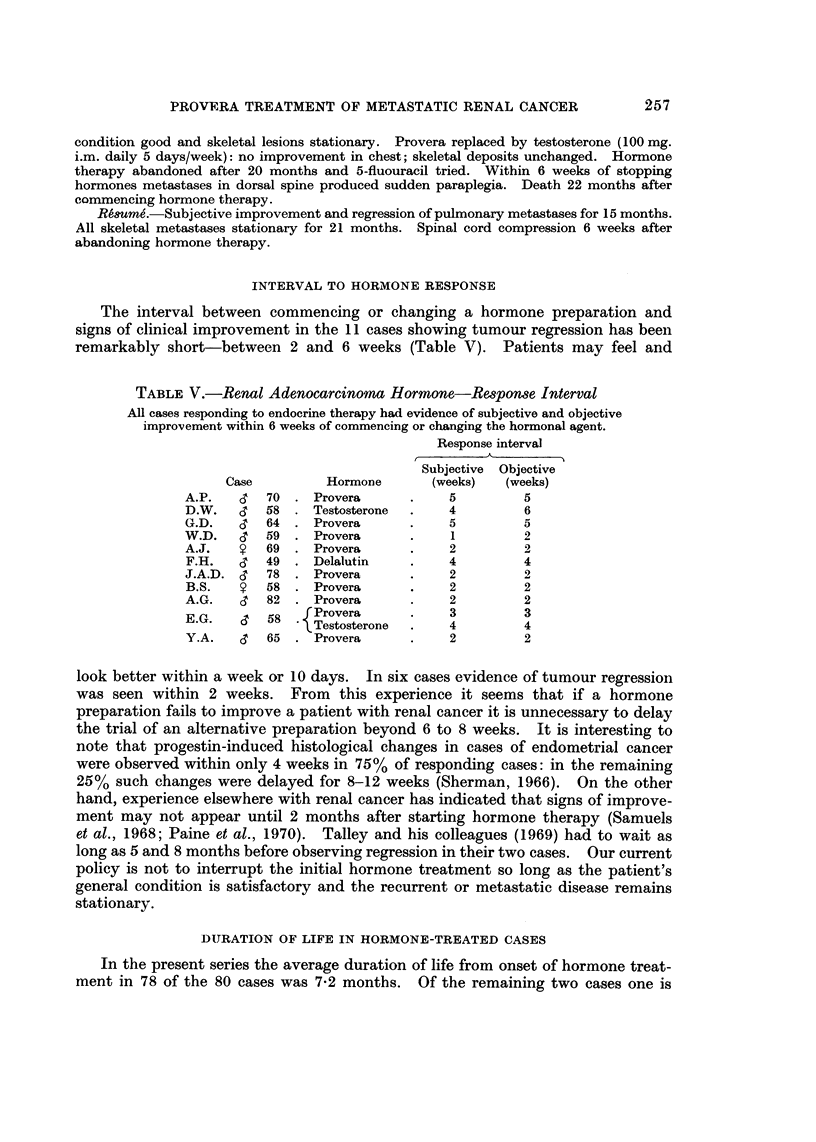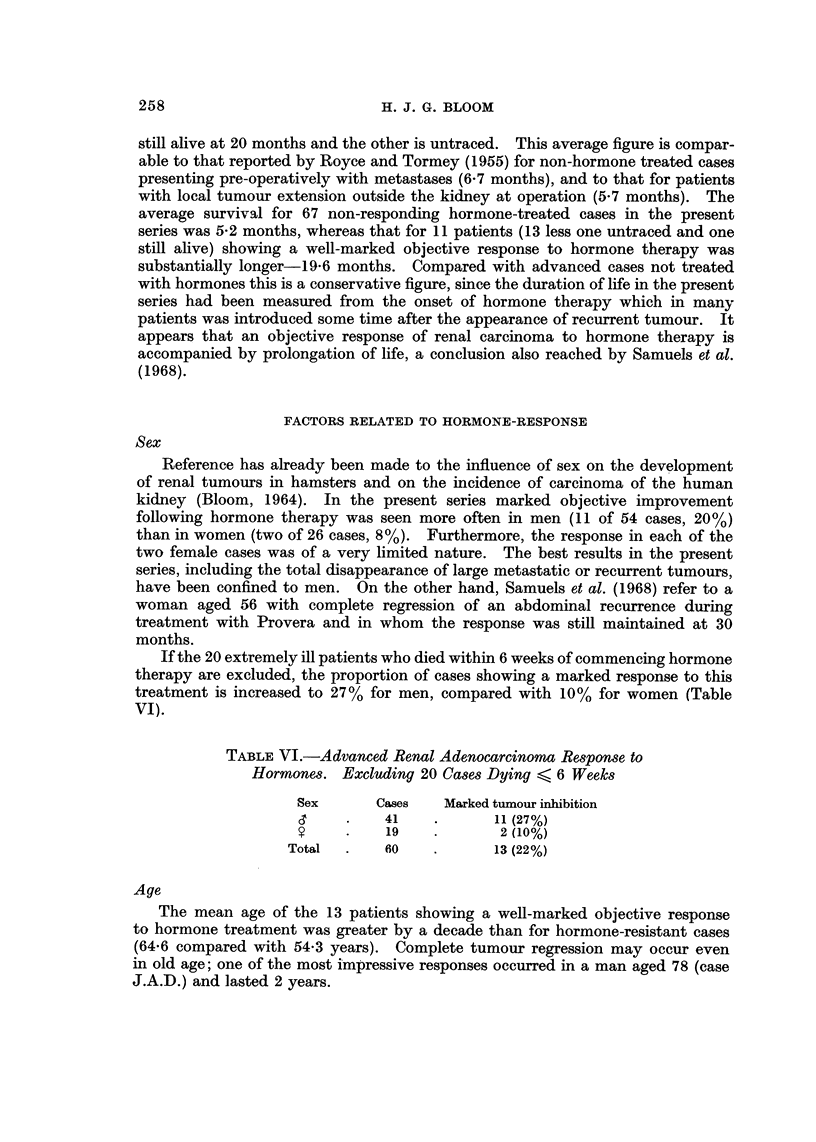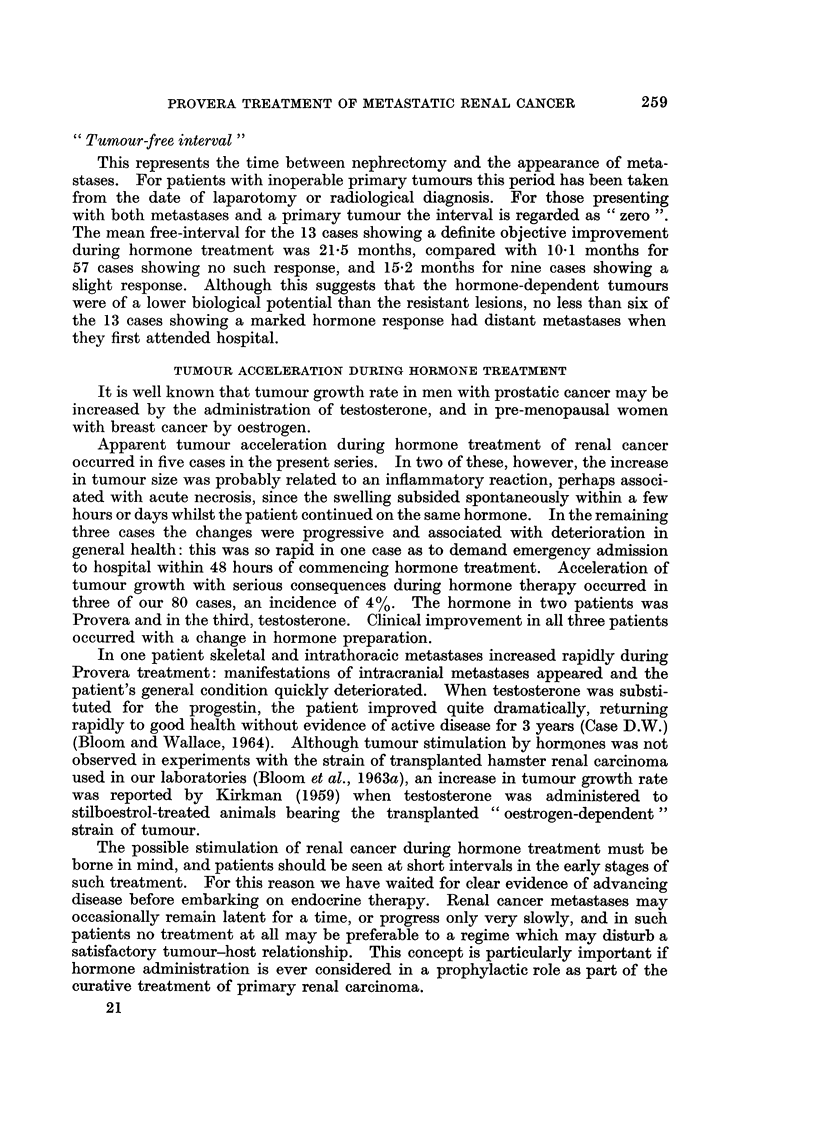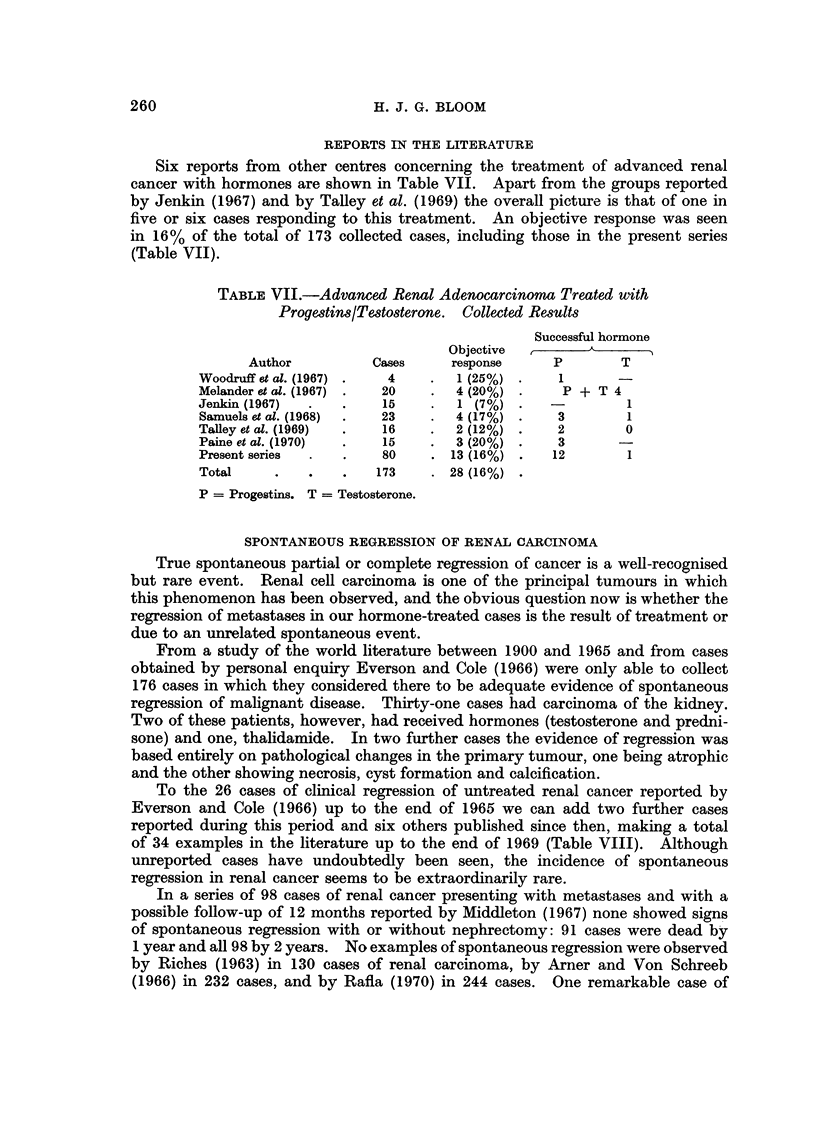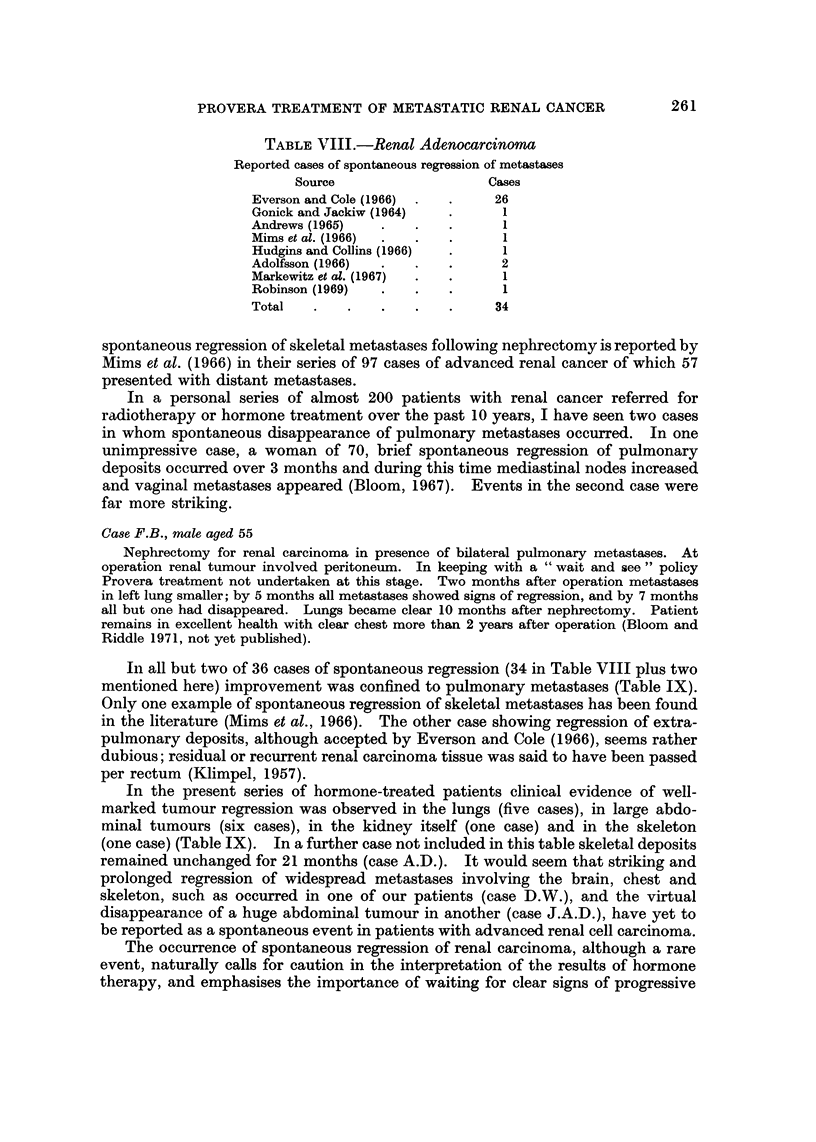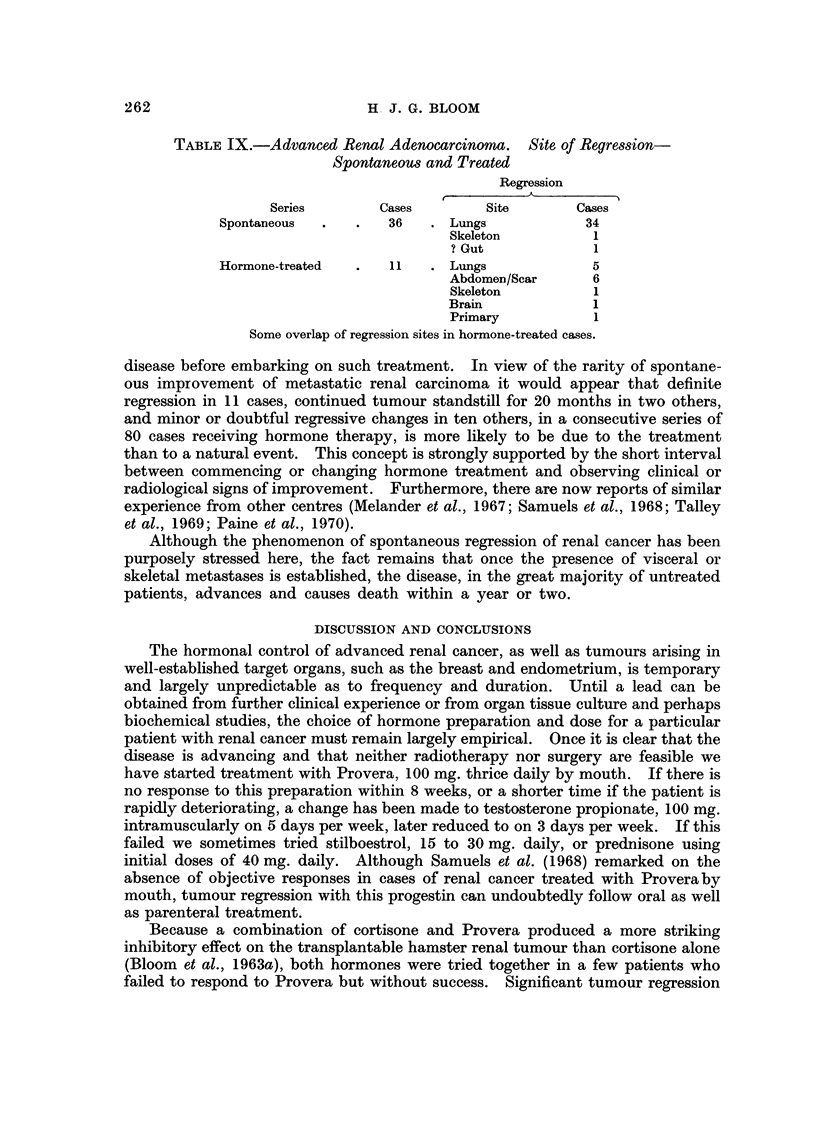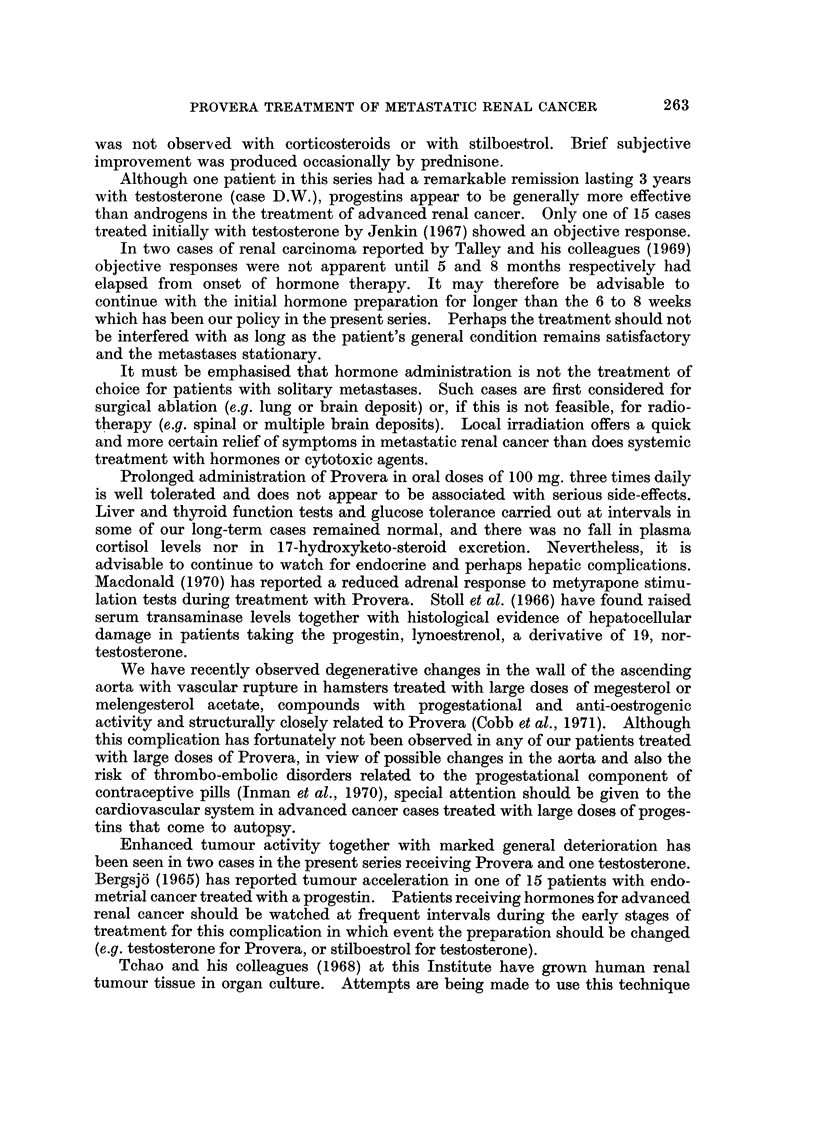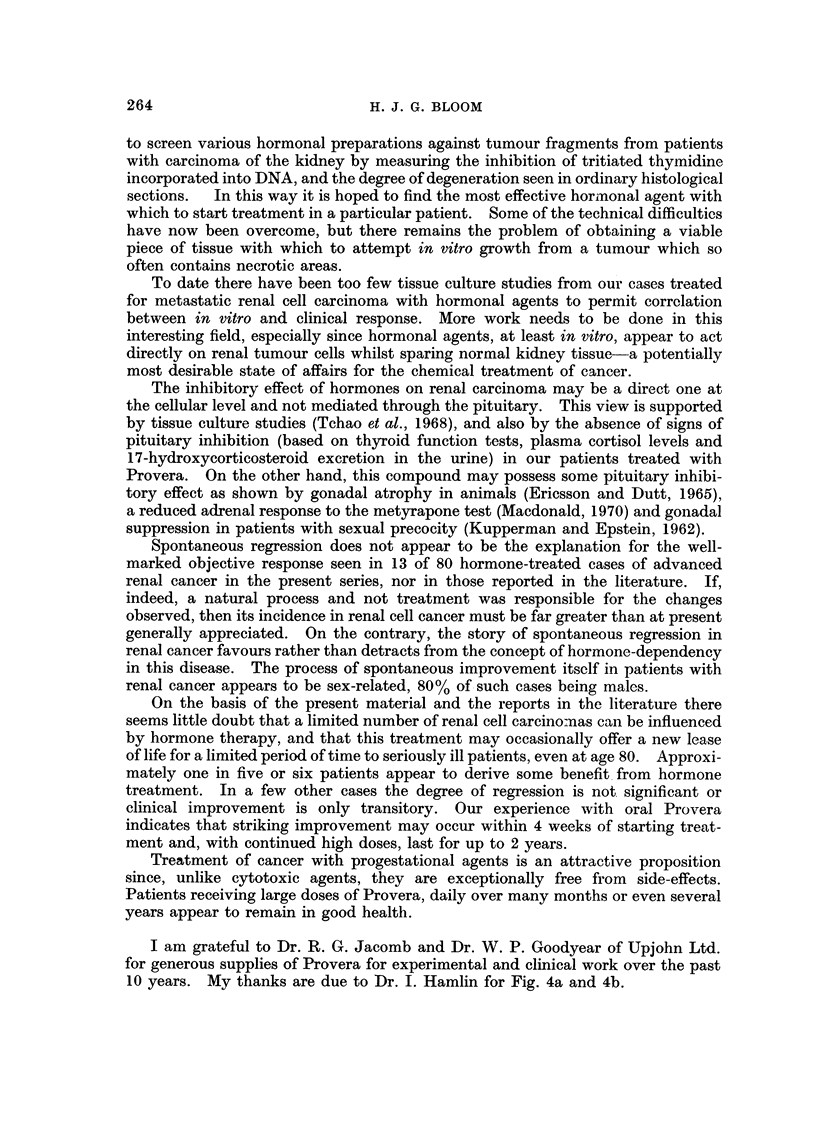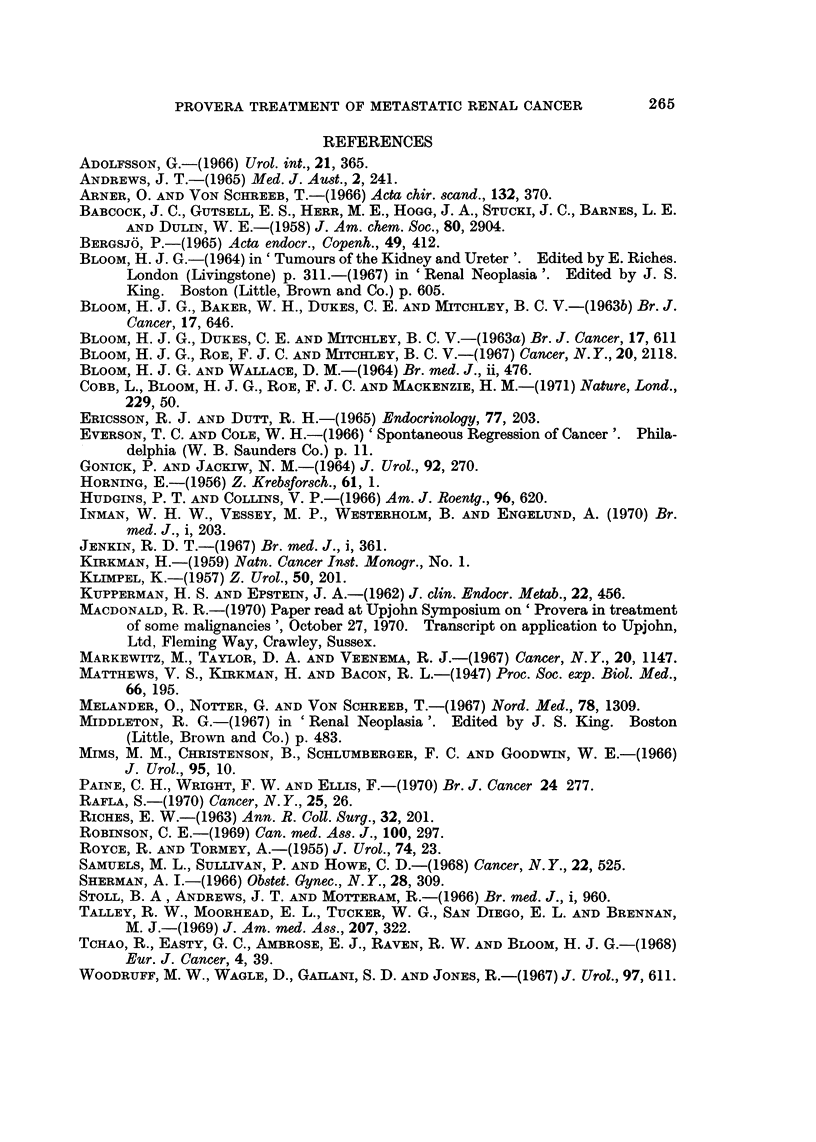# Medroxyprogesterone Acetate (Provera) in the Treatment of Metastatic Renal Cancer*

**DOI:** 10.1038/bjc.1971.31

**Published:** 1971-06

**Authors:** H. J. G. Bloom

## Abstract

**Images:**


### MEDROXYPROGESTERONE ACETATE (PROVERA) IN THE TREATMENT OF METASTATIC RENAL CANCER*

H. J. G. BLOOM

*From the Royal Marsden Hospital and Institute of Cancer Research, London, S.W.3, and the St. Peter's Group of Hospitals, London, W.C.2*

Received for publication April 30, 1971

**SUMMARY.—Eighty patients with advanced metastatic renal cancer have been treated with hormones, chiefly medroxyprogesterone acetate (Provera). This progestational compound is remarkably free from side-effects and can be given in high dosage for long periods without serious complications. Ninety per cent of cases had multiple metastases: in 76% more than one organ was involved and nearly 50% were seriously ill or " terminal ".**

**Subjective improvement occurred in at least 55%. In 11 patients there was marked improvement in the radiological or clinical signs of tumour within 2 to 6 weeks of commencing treatment or changing to a different hormone. In two further cases improved general health was associated with stationary metastases for 20 months. A significant objective response occurred in 16% of the total series. A favourable response was seen more often in men (21%) than in women (8%). If deaths within 6 weeks are excluded the objective response rate in men is increased to 27%. Although the response of advanced renal cancer to hormonal treatment is usually incomplete and of brief duration, it is possible for such treatment to offer a " new lease of life " to a seriously ill patient, even in old age, for 2 to 3 years.**

BETWEEN 75% and 80% of patients treated for renal cell carcinoma will die within 10 years of their primary treatment, the majority with distant metastases. In a few of these cases the natural history of the disease will be unusual. For example, growth of metastases may be exceptionally slow or, following removal of a solitary lesion, the patient may live for a number of years without further recurrence. Very rarely spontaneous regression of pulmonary deposits may occur. For the vast majority of cases, however, the onset of clinical metastases heralds death within a year or two.

The treatment of advanced renal cancer with cytotoxic drugs has been disappointing. Woodruff *et al.* (1967) reviewed the literature on this subject, and of 243 collected cases treated with 33 different non-hormonal agents only 10% showed signs of objective improvement: in most instances this was slight or of brief duration. Talley *et al.* (1969) have published their personal experience with various cytotoxic drugs against metastatic hypernephroma. Of 57 patients treated with 15 different non-hormonal compounds, chiefly alkylating agents, antimetabolites and vinblastine, objective improvement was seen in only two cases (3·5%). Apart from these poor results, the agents employed are highly toxic and have a relatively small margin of safety.

* Based on a paper read at the Upjohn Symposium on " Provera in Treatment of Some Malignancies ", The Royal Society of Medicine, October 27, 1970.

In previous publications we have drawn attention to clinical, pathological and experimental evidence in support of the concept of a hormonal background to carcinoma of the kidney, and to the possibility of achieving worthwhile palliation in a small proportion of very advanced cases by the administration of gonadal hormones, chiefly progestins (Bloom *et al.*, 1963*a* 1963*b*; Bloom and Wallace, 1964; Bloom, 1964, 1967). Such agents appear to be not only more successful in controlling the disease than cytotoxic drugs, but are also remarkably free from serious side-effects following prolonged administration in large doses.

A transplantable renal adenocarcinoma of the golden hamster, induced by prolonged oestrogen administration (Matthews *et al.*, 1947; Kirkman, 1959; Horning, 1956), has been employed as an experimental model with which to try and find hormonal agents which may be of value in the treatment of advanced renal cancer in man (Bloom *et al.*, 1963*a*, 1967).

Primary tumour induction in the hamster by oestrogen can be inhibited by the simultaneous administration of testosterone or progesterone (Kirkman, 1959; Horning, 1956). Provera combined with cortisone, but not alone, markedly reduced the growth rate of " independent " tumour transplants (Bloom *et al.*, 1963*a*). Actual tumour regression was achieved by orchiectomy and this action was nullified by the administration of oestradiol (Bloom *et al.*, 1963*b*). For this reason the effect of anti-oestrogens was investigated and the first of these studied, U-11100A (Upjohn Ltd.), a diphenyldihydronaphthaline derivative, had a marked inhibitory effect on the transplantable tumour (Bloom *et al.*, 1967). Other agents, including those with known anti-oestrogenic properties, are under investigation in our laboratories at the present time.

Caution is necessary when trying to extrapolate from observations in laboratory animals to clinical practice, but because the principal actions of hormonal agents are fundamentally alike in most species, it is tempting to apply knowledge derived from endocrine-dependent tumours in animals to possibly analogous tumours in man. Although the kidney is not generally regarded as a member of the endocrine system, a direct link between hormones and human renal cancer exists in the rare but well-recognised association of renal cell carcinoma with polycythaemia and with hypercalcoemia due to the " ectopic " secretion of erythropoietin and parathormone, respectively, by the tumour.

The male predominance found in renal cortical carcinoma series and in cases of spontaneous regression of this tumour, the effect of sex hormones on the normal kidney in experimental animals, and the induction and inhibition of the hamster renal tumour by such agents, encouraged us in 1959 to embark on a clinical trial of gonadal hormones, chiefly the progestin, provera, in patients with advanced renal cancer (Bloom, 1964).

### PATIENTS AND METHODS

Between 1959 and 1969 80 patients with incurable renal adenocarcinoma have been treated with hormonal agents. Only cases with clear evidence of advancing disease for whom no other treatment, except by cytotoxic drugs, was feasible were accepted for hormone therapy. In this category no case was refused treatment, no matter how near death they appeared to be. Thus, 20 very ill patients died within 6 weeks of commencing hormone treatment (25% of the total).

The series consisted of 54 men and 26 women. A nephrectomy had been carried out in 67 patients and histological confirmation of the diagnosis of renal carcinoma was obtained in 73 (91%). Of the 80 cases 94% had multiple metastases and in 76% more than one organ was known to be involved (Table I). Thirty-eight cases (47%) were seriously ill or considered to be "terminal" when hormone therapy was instituted. In 19 cases (24%), the liver was enlarged, in eight as far as the umbilicus. Seven patients had cerebral metastases. Skeletal deposits were present in 23 (29%) cases, and pulmonary or mediastinal lesions in 57 (71%). A laparotomy in seven patients revealed that proper removal of the primary tumour was impossible (Table I).

TABLE I.—*Cases of Advanced Renal Adenocarcinoma Treated with Hormones.* 80 *Cases* (1959–69)

| Site | Cases | % |
|---|---|---|
| Multiple metastases | 75 | 94 |
| Multiple different organs involved | 61 | 76 |
| Lungs, mediastinum | 57 | 71 |
| Skeleton | 23 | 29 |
| Hepatomegaly* | 19 | 24 |
| Brain | 7 | 9 |
| Inoperable primary† | 10 | 12 |
| Other abdominal masses‡ | 17 | 21 |
| Seriously ill or terminal | 38 | 47 |
| Presented primary + metastases | 32 | 40 |

* Umbilical level in 8; † Laparotomy in 7; ‡ > 12 cm. in 9.

Treatment in all cases was initiated with progestins—Provera in 79 cases and Delalutin in one case. Provera or medroxypyrogesterone acetate (6α-methyl, 17α-acetoxy-progesterone) is a powerful progestational agent synthesized in the laboratories of Upjohn Ltd (Babock *et al.*, 1958) which is devoid of oestrogenic and androgenic properties. It may be given orally or as a suspension intramuscularly. Prolonged administration to patients even in high doses (300–400 mg. daily) appears to be virtually free from serious toxic effects. In the present series the oral preparation of Provera was used 100 mg. t.i.d. Delalutin or Primolut Depot (Schering) (hydroxyprogesterone caproate) was given in a dose of 250 mg. i.m. thrice weekly.

PROGESTERONE

PROVERA (Medroxyprogesterone acetate)

RESULTS

Of the 80 cases 78 have been followed until death; one foreign patient remains untraced and one case is still alive. A significant degree of subjective improvement was experienced by 44 patients (55%) following the administration of one or more hormone preparations. Attention will be given only to cases with objective signs of tumour regression or where the tumour remained stationary for more than 1 year. In this category ten cases thought to show some response have been mentioned but excluded from the final consideration because the change was of a very limited nature, lasted for 1 month or less, or was not confirmed by the present author.

Eleven cases (14%) remain in which undeniable improvement in the radiological or clinical signs of tumour occurred within a short time of commencing treatment or changing to a different hormone and lasted for 2–35 months (Tables II and III). There were two additional cases in whom marked improvement in general condition was associated with stationary tumour for 20 and 21 months respectively (Table IV). Histological confirmation of the diagnosis was obtained in all 13 cases, and in five of the 11 dead cases an autopsy was performed.

TABLE II.—*Advanced Renal Adenocarcinoma. Results of Hormone Treatment.* 80 *Cases*

| Response | Cases | % |
|---|---|---|
| Subjective | 44 | 55 |
| Objective | | |
| Marked tumour regression | 11* | 14 |
| Tumour stationary > 1 year | 2† | 2·5 |
| Slight tumour regression | 10 | 12 |
| Total objective | 23 | 29 |

* For 2, 2, 3, 3, 3, 9, 13, 20, 24, 24, 35 months.
† For 21, 20+ months.

TABLE III.—*Hormone Therapy for Metastatic Renal Cancer Marked Objective Response*

The organs in which tumour regression occurred are underlined. In some patients not all known tumour sites responded to treatment.

| Case | Sex age | Extent prior to hormones | Successful hormone | Duration of response (months) | Survival from start of hormones (months) |
|---|---|---|---|---|---|
| A.P. | ♂ 70 | Lung, bone | Provera | 20 | 32 |
| D.W. | ♂ 58 | Lung, bone, brain | Testosterone | 35 | 41 |
| G.D. | ♂ 64 | Lung | Provera | 9 | 13 |
| W.D. | ♂ 59 | Liver, lung, inop. primary | Provera | 3 | 3 |
| A.J. | ♀ 69 | Abdominal masses | Provera | 3 | 7 |
| F.H. | ♂ 49 | Abdomen, suprac. nodes | Delalutin / Testosterone | 22 | 24 |
| J.A.D. | ♂ 78 | Lung, scar | Provera | 24 | 37 |
| B.S. | ♀ 58 | Inop. primary, liver | Provera | 2 | 3 |
| A.G. | ♂ 82 | Scar, abdomen | Provera | 3 | 14 |
| E.G. | ♂ 58 | Lungs, abdomen | Provera / Testosterone | 9 / 4 | 20 |
| Y.A. | ♂ 65 | Scar, abdomen | Provera | 2·5 | Untraced |

The overall significant objective response rate is 16% (13 of 80 cases). If the 20 seriously ill patients who died within 6 weeks of starting hormone therapy are excluded on the grounds of insufficient time for a chemotherapeutic effect, a practice endorsed by several authors, the response rate is 22% (Table VI).

TABLE IV.—*Disease Stationary for* $>12$ *Months*

| Case | Sex age | Extent prior to hormones | Hormone | Response | Survival |
|---|---|---|---|---|---|
| A.D. | ♂ 71 | Bone, lung | Provera | Multiple bone mets. stationary 21 months | Died 22 months: lung and spinal mets. active. |
| | | | Testosterone | Regression lung mets. 15 months | |
| W.G. | ♂ 62 | Mediastinum | Provera | Mass stationary 20+ months | Alive and well 20 months |

ILLUSTRATIVE CASE SUMMARIES

*Partial Selective Regression*

*Case A.P., male aged* 70 (*Bloom*, 1964)

Metastases in left lung and lower femur 4 years after nephrectomy. Oral Provera, 300 mg. daily, prescribed and irradiation to femoral deposit because of pain and risk of fracture. Six weeks later large lung deposit smaller but femoral lesion advancing. By 4 months mid-thigh amputation for pathological fracture. Pulmonary metastasis continued to regress and finally became stationary: this response maintained for 20 months. Progressive destruction of femoral stump required hip disarticulation. Intrathoracic metastases recurred with no response to larger doses of Provera nor to prednisone, Provera plus prednisone and finally testosterone. Death with wide-spread metastases 32 months after starting hormone therapy.

*Résumé.*—Partial regression of large pulmonary metastasis commencing within 5 weeks and lasting 20 months: no response of skeletal deposit which advanced during treatment.

*Case B.S., female aged* 58 (*Bloom*, 1967)

General condition very poor, massive tumour in solitary kidney and hard irregular liver to umbilicus. Nephrectomy for tuberculosis 32 years previously. Recent excision of thyroid mass which showed metastatic clear cell adenocarcinoma. Nephrotomogram revealed small cap of functioning renal tissue atop a large renal tumour. Oral Provera 300 mg. daily started. After 10 days improved general condition, increased appetite, weight gain, more physical activity with less fatigue and renal mass smaller: hepatomegaly unchanged. Discharged from hospital after 6 weeks with renal tumour approximately one-third of its original size, but liver unchanged. General improvement and response of primary tumour maintained for 2 months after which there was deterioration and increasing hepatomegaly. No response to testosterone. Death 3 months after commencing hormone therapy. Autopsy: large renal tumour showing extensive areas of necrosis and calcification. Histology: residual clear cell adenocarcinoma and extensive areas of anaplastic tumour with marked pleomorphism and hyperchromatism.

*Résumé.*—Rapid partial regression of massive primary tumour. Unchanged metastatic liver. Was the partial response limited to the areas of more differentiated tumour?

*Total Selective Regression*

*Case G.D., male aged* 64 (*Bloom*, 1964)

Presented with renal tumour and solitary pulmonary shadow. Pre-operative irradiation for primary tumour during which time pulmonary deposit increased. Nephrectomy carried out. Two months later chest X-ray showed numerous bilateral lung lesions. Oral Provera, 300 mg. daily, started. Five weeks later pulmonary deposits reduced in size and number, and after 2 months all but two lesions had disappeared. These remained stationary for 8 months during which time patient was symptom-free and travelled abroad. Metastases then advanced with no response to prednisone or testosterone. Urinary complications developed suggesting metastases in remaining kidney. Died at home 13 months after starting hormone therapy.

*Résumé.*—Disappearance of all but two metastases in lung within 8 weeks. Response lasted 9 months.

*Case W.D., male aged* 59 (*Bloom*, 1964)

Huge inoperable renal tumour and bilateral pulmonary metastases. During pre-operative irradiation to primary tumour, pulmonary lesions increased. At laparotomy (Sir Eric Riches) large fixed kidney tumour with deposits in liver, para-aortic nodes and diaphragm. Biopsy of diaphragmatic nodule confirmed renal cell carcinoma. Post-operatively, general condition deteriorated further and pulmonary deposits and abdominal swelling increased. Oral Provera, 300 mg. daily, started. Six days later patient felt and looked rather better. By 13 days definite improvement in appearance and some regression of pulmonary metastases. Discharged home 15 days after starting hormone therapy. At 8 weeks lung clear but abdomen still distended by large liver and ascites. Death at home 3 months after starting Provera.

*Résumé.*—Clinical improvement within 1 week; objective response within 2 weeks. Complete disappearance of pulmonary metastases within 8 weeks but no change in hepatomegaly.

*Total Regression*

*Case J.A.D., male aged* 78

General weakness, chest and abdominal symptoms 9 months after nephrectomy. Abdominal mass 15 × 12 cm. involving nephrectomy scar (Fig. 1a): second more superficial mass 8 × 6 cm. in drain scar (Fig. 1b). Multiple small bilateral lung metastases (Fig. 2a). Marked subjective and objective response within 2 weeks of commencing oral Provera 300 mg. daily. By 4 weeks larger abdominal tumour reduced to 6 × 6 cm. and smaller to 2·5 cm. At 8 weeks chest clear (Fig. 2b) and total regression of smaller abdominal recurrence. Larger abdominal tumour now a superficial lump 3·5 cm. (Fig. 1c). General condition good; symptom-free; able to walk 2 miles daily.

At 3 months excision of residual abdominal tumour 2·5 cm. (Fig. 3). Histology: secondary renal cell carcinoma (Fig. 4a), appearance suggesting lower grade activity than original primary (Fig. 4b).

Patient passed 80th birthday in good health (Fig. 5b). Abdominal recurrence 25 months after starting Provera which progressed slowly over 12 months during successive administration of larger doses of Provera (400 mg. daily), an anti-oestrogen (U11-100A) and testosterone. Death aged 81, 3 years after commencing hormone therapy.

*Résumé.*—Rapid total disappearance of lung metastases and scar recurrence. Sub-total regression of huge abdominal tumour. Benefit lasted 2 years.

*Tumour Acceleration with Provera: Total Regression with Testosterone*

*Case D.W., male aged* 58 (*Bloom and Wallace*, 1964)

Presented with renal tumour and metastases in tibia, chest, and skull. Nephrectomy (D. M. Wallace). Because of pain and risk of fracture large osteolytic deposit upper tibia treated by irradiation, curretage, bone-chip replacement and insertion of intramedullary nail (G. R. Fisk). Whilst receiving oral Provera 300 mg. daily over 2 months marked general deterioration, onset of hemiparesis and increase of intrathoracic and unirradiated skeletal metastases. Provera replaced by 100 mg. testosterone i.m. daily, 5 days weekly. Within 4 weeks striking improvement in general health; by 8 weeks described as a " new man ".

After 18 months on testosterone patient was in good health and full employment: weight gain 23 kg., recovery from hemiparesis. Radiological regression of metastases in skull and tibia (unirradiated area). Surgically treated and irradiated tibial metastasis healed. Chest clear. Continued in good health with no evidence of active disease for 3 years. Then sudden collapse with hemiplegia. No recurrence of skeletal lesions but new soft tissue metastases. Stilboestrol, prednisone and further testosterone all tried in turn without effect. Death with visceral metastases 41 months after starting hormone therapy. At autopsy, previous large skull defect covered by fibrous membrane with no evidence of residual tumour. (Full illustrated account of this case in British Medical Journal, August 22, 1964, pp. 476–480).

*Résumé.*—Tumour acceleration with Provera. Rapid improvement on changing to testosterone. Total regression of chest and skeletal metastases for 35 months.

### *Partial Selective Regression with Two Separate Hormones*

*Case E.G., male aged* 58

Pulmonary deposit and abdominal mass with scar involvement 6 years after nephrectomy. Gradual increase of pulmonary lesion over 12 months whilst abdominal mass remained quiescent following local irradiation (4000 R). Returned with painful abdominal tumour 9 × 10 cm. and large lung mass. Oral Provera 300 mg. daily started. Within 3 weeks pain-free and reduction of abdominal mass to 7 × 7 cm. At 6 weeks, improved general condition and weight gain 2·5 kg. By 12 weeks abdominal tumour stationary at 5 × 5 cm.: lung deposit unchanged throughout. This situation continued for 10 months from onset of Provera. Then abdominal tumour increased to 14 × 13 cm. within 8 weeks. Provera replaced by testosterone, initially 100 mg. i.m. daily, 5 days weekly. Second subjective and objective response, abdominal tumour shrinking to 8 × 8 cm. within 4 weeks. General condition remained excellent, lung deposit unchanged, and abdominal mass stationary at 4 × 4 cm. After 4 months chest lesion advanced producing mediastinal obstruction which was treated by irradiation. Abdominal tumour still stationary. General deterioration with death at home, 20 months after commencing hormones.

*Résumé*.—Rapid subjective improvement and partial regression of abdominal tumour, first with Provera and later with testosterone. No change in pulmonary mass with either agent.

### *Tumour " Stand-still "*

*Case W.G., male aged* 62

Renal tumour and superior mediastinal mass. Nephrectomy; tumour invading posterior abdominal wall and para-aortic region. General weakness, poor appetite and weight loss. Oral Provera 300 mg. daily started because of known residual disease in abdomen and the mediastinal tumour, presumed lymph node metastases. Within 15 days felt better, appetite improved and weight increased by 2·25 kg. At 12 months general condition good, total weight gain 13·5 kg. Alive and well 20 months after commencing Provera: mediastinal mass completely unchanged; no new lesions.

*Résumé*.—Improved general condition, stationary mediastinal mass and no recurrence of known post-operative residual abdominal disease at 20 months.

*Case A.D., male, aged* 67

Widespread skeletal metastases involving cervical and dorsal spine, skull, humeri, pelvis and femora 4 years after nephrectomy. Also two deposits in left lung. Oral Provera 300 mg. daily started, but because of risk of quadriplegia due to collapse of C4, cervical spine irradiated. Marked general improvement with weight gain. After 3 months lungs practically clear. Radiological evidence of healing in cervical spine (irradiated). All other osteolytic skeletal deposits unchanged. At 15 months recurrence of disease in left lung but general

---

### EXPLANATION OF PLATES

FIG. 1a.—Case J.A.D. Large abdominal recurrence (15 × 12 cm.) 9 months after nephrectomy for renal carcinoma.
FIG. 1b.—Case J.A.D. Drainage scar recurrence 8 × 6 cm.
FIG. 1c.—Case J.A.D. Regression of abdominal tumour to 3·5 cm. and disappearance of drainage recurrence 8 weeks after starting oral Provera 300 mg. daily.
FIG. 2a.—Case J.A.D. Multiple bilateral pulmonary metastases.
FIG. 2b.—Case J.A.D. Disappearance of all pulmonary metastases after 8 weeks treatment with Provera.
FIG. 3.—Residual abdominal tumour 2·5 cm. excised after 12 weeks treatment with Provera.
FIG. 4a.—Histology of residual tumour seen in Fig. 3 (×300). Renal carcinoma deposit appearing less active (lower grade) than original primary tumour in Fig. 4b.
FIG. 4b.—Renal cell carcinoma—nephrectomy specimen (×300).
FIG. 5a.—Patient J.A.D. one month after commencing oral Provera for extensive pulmonary and abdominal recurrent renal carcinoma.
FIG. 5b.—Case J.A.D. Aged 80, clinically and radiologically still free from disease after receiving Provera for 15 months. Excellent response maintained for 2 years.

1a

1b

1c

2a

2b

Bloom

3

4a

4b

Bloom

5a

5b

Bloom condition good and skeletal lesions stationary. Provera replaced by testosterone (100 mg. i.m. daily 5 days/week): no improvement in chest; skeletal deposits unchanged. Hormone therapy abandoned after 20 months and 5-fluouracil tried. Within 6 weeks of stopping hormones metastases in dorsal spine produced sudden paraplegia. Death 22 months after commencing hormone therapy.

*Résumé.*—Subjective improvement and regression of pulmonary metastases for 15 months. All skeletal metastases stationary for 21 months. Spinal cord compression 6 weeks after abandoning hormone therapy.

### INTERVAL TO HORMONE RESPONSE

The interval between commencing or changing a hormone preparation and signs of clinical improvement in the 11 cases showing tumour regression has been remarkably short—between 2 and 6 weeks (Table V). Patients may feel and

TABLE V.—*Renal Adenocarcinoma Hormone—Response Interval*

All cases responding to endocrine therapy had evidence of subjective and objective improvement within 6 weeks of commencing or changing the hormonal agent.

| Case | | | Hormone | Response interval: Subjective (weeks) | Objective (weeks) |
|---|---|---|---|---|---|
| A.P. | ♂ | 70 | Provera | 5 | 5 |
| D.W. | ♂ | 58 | Testosterone | 4 | 6 |
| G.D. | ♂ | 64 | Provera | 5 | 5 |
| W.D. | ♂ | 59 | Provera | 1 | 2 |
| A.J. | ♀ | 69 | Provera | 2 | 2 |
| F.H. | ♂ | 49 | Delalutin | 4 | 4 |
| J.A.D. | ♂ | 78 | Provera | 2 | 2 |
| B.S. | ♀ | 58 | Provera | 2 | 2 |
| A.G. | ♂ | 82 | Provera | 2 | 2 |
| E.G. | ♂ | 58 | Provera | 3 | 3 |
| | | | Testosterone | 4 | 4 |
| Y.A. | ♂ | 65 | Provera | 2 | 2 |

look better within a week or 10 days. In six cases evidence of tumour regression was seen within 2 weeks. From this experience it seems that if a hormone preparation fails to improve a patient with renal cancer it is unnecessary to delay the trial of an alternative preparation beyond 6 to 8 weeks. It is interesting to note that progestin-induced histological changes in cases of endometrial cancer were observed within only 4 weeks in 75% of responding cases: in the remaining 25% such changes were delayed for 8–12 weeks (Sherman, 1966). On the other hand, experience elsewhere with renal cancer has indicated that signs of improvement may not appear until 2 months after starting hormone therapy (Samuels *et al.*, 1968; Paine *et al.*, 1970). Talley and his colleagues (1969) had to wait as long as 5 and 8 months before observing regression in their two cases. Our current policy is not to interrupt the initial hormone treatment so long as the patient's general condition is satisfactory and the recurrent or metastatic disease remains stationary.

### DURATION OF LIFE IN HORMONE-TREATED CASES

In the present series the average duration of life from onset of hormone treatment in 78 of the 80 cases was 7·2 months. Of the remaining two cases one is still alive at 20 months and the other is untraced. This average figure is comparable to that reported by Royce and Tormey (1955) for non-hormone treated cases presenting pre-operatively with metastases (6·7 months), and to that for patients with local tumour extension outside the kidney at operation (5·7 months). The average survival for 67 non-responding hormone-treated cases in the present series was 5·2 months, whereas that for 11 patients (13 less one untraced and one still alive) showing a well-marked objective response to hormone therapy was substantially longer—19·6 months. Compared with advanced cases not treated with hormones this is a conservative figure, since the duration of life in the present series had been measured from the onset of hormone therapy which in many patients was introduced some time after the appearance of recurrent tumour. It appears that an objective response of renal carcinoma to hormone therapy is accompanied by prolongation of life, a conclusion also reached by Samuels *et al.* (1968).

### FACTORS RELATED TO HORMONE-RESPONSE

### *Sex*

Reference has already been made to the influence of sex on the development of renal tumours in hamsters and on the incidence of carcinoma of the human kidney (Bloom, 1964). In the present series marked objective improvement following hormone therapy was seen more often in men (11 of 54 cases, 20%) than in women (two of 26 cases, 8%). Furthermore, the response in each of the two female cases was of a very limited nature. The best results in the present series, including the total disappearance of large metastatic or recurrent tumours, have been confined to men. On the other hand, Samuels *et al.* (1968) refer to a woman aged 56 with complete regression of an abdominal recurrence during treatment with Provera and in whom the response was still maintained at 30 months.

If the 20 extremely ill patients who died within 6 weeks of commencing hormone therapy are excluded, the proportion of cases showing a marked response to this treatment is increased to 27% for men, compared with 10% for women (Table VI).

TABLE VI.—*Advanced Renal Adenocarcinoma Response to Hormones. Excluding* 20 *Cases Dying* $\leqslant$ 6 *Weeks*

| Sex | Cases | Marked tumour inhibition |
|---|---|---|
| ♂ | 41 | 11 (27%) |
| ♀ | 19 | 2 (10%) |
| Total | 60 | 13 (22%) |

### *Age*

The mean age of the 13 patients showing a well-marked objective response to hormone treatment was greater by a decade than for hormone-resistant cases (64·6 compared with 54·3 years). Complete tumour regression may occur even in old age; one of the most impressive responses occurred in a man aged 78 (case J.A.D.) and lasted 2 years.

*" Tumour-free interval "*

This represents the time between nephrectomy and the appearance of metastases. For patients with inoperable primary tumours this period has been taken from the date of laparotomy or radiological diagnosis. For those presenting with both metastases and a primary tumour the interval is regarded as " zero ". The mean free-interval for the 13 cases showing a definite objective improvement during hormone treatment was 21·5 months, compared with 10·1 months for 57 cases showing no such response, and 15·2 months for nine cases showing a slight response. Although this suggests that the hormone-dependent tumours were of a lower biological potential than the resistant lesions, no less than six of the 13 cases showing a marked hormone response had distant metastases when they first attended hospital.

### TUMOUR ACCELERATION DURING HORMONE TREATMENT

It is well known that tumour growth rate in men with prostatic cancer may be increased by the administration of testosterone, and in pre-menopausal women with breast cancer by oestrogen.

Apparent tumour acceleration during hormone treatment of renal cancer occurred in five cases in the present series. In two of these, however, the increase in tumour size was probably related to an inflammatory reaction, perhaps associated with acute necrosis, since the swelling subsided spontaneously within a few hours or days whilst the patient continued on the same hormone. In the remaining three cases the changes were progressive and associated with deterioration in general health: this was so rapid in one case as to demand emergency admission to hospital within 48 hours of commencing hormone treatment. Acceleration of tumour growth with serious consequences during hormone therapy occurred in three of our 80 cases, an incidence of 4%. The hormone in two patients was Provera and in the third, testosterone. Clinical improvement in all three patients occurred with a change in hormone preparation.

In one patient skeletal and intrathoracic metastases increased rapidly during Provera treatment: manifestations of intracranial metastases appeared and the patient's general condition quickly deteriorated. When testosterone was substituted for the progestin, the patient improved quite dramatically, returning rapidly to good health without evidence of active disease for 3 years (Case D.W.) (Bloom and Wallace, 1964). Although tumour stimulation by hormones was not observed in experiments with the strain of transplanted hamster renal carcinoma used in our laboratories (Bloom *et al.*, 1963*a*), an increase in tumour growth rate was reported by Kirkman (1959) when testosterone was administered to stilboestrol-treated animals bearing the transplanted " oestrogen-dependent " strain of tumour.

The possible stimulation of renal cancer during hormone treatment must be borne in mind, and patients should be seen at short intervals in the early stages of such treatment. For this reason we have waited for clear evidence of advancing disease before embarking on endocrine therapy. Renal cancer metastases may occasionally remain latent for a time, or progress only very slowly, and in such patients no treatment at all may be preferable to a regime which may disturb a satisfactory tumour–host relationship. This concept is particularly important if hormone administration is ever considered in a prophylactic role as part of the curative treatment of primary renal carcinoma.

### REPORTS IN THE LITERATURE

Six reports from other centres concerning the treatment of advanced renal cancer with hormones are shown in Table VII. Apart from the groups reported by Jenkin (1967) and by Talley *et al.* (1969) the overall picture is that of one in five or six cases responding to this treatment. An objective response was seen in 16% of the total of 173 collected cases, including those in the present series (Table VII).

TABLE VII.—*Advanced Renal Adenocarcinoma Treated with Progestins/Testosterone. Collected Results*

| | | | Successful hormone | |
|---|---|---|---|---|
| Author | Cases | Objective response | P | T |
| Woodruff *et al.* (1967) | 4 | 1 (25%) | 1 | — |
| Melander *et al.* (1967) | 20 | 4 (20%) | P + T 4 | |
| Jenkin (1967) | 15 | 1 (7%) | — | 1 |
| Samuels *et al.* (1968) | 23 | 4 (17%) | 3 | 1 |
| Talley *et al.* (1969) | 16 | 2 (12%) | 2 | 0 |
| Paine *et al.* (1970) | 15 | 3 (20%) | 3 | — |
| Present series | 80 | 13 (16%) | 12 | 1 |
| Total | 173 | 28 (16%) | | |

P = Progestins. T = Testosterone.

### SPONTANEOUS REGRESSION OF RENAL CARCINOMA

True spontaneous partial or complete regression of cancer is a well-recognised but rare event. Renal cell carcinoma is one of the principal tumours in which this phenomenon has been observed, and the obvious question now is whether the regression of metastases in our hormone-treated cases is the result of treatment or due to an unrelated spontaneous event.

From a study of the world literature between 1900 and 1965 and from cases obtained by personal enquiry Everson and Cole (1966) were only able to collect 176 cases in which they considered there to be adequate evidence of spontaneous regression of malignant disease. Thirty-one cases had carcinoma of the kidney. Two of these patients, however, had received hormones (testosterone and prednisone) and one, thalidamide. In two further cases the evidence of regression was based entirely on pathological changes in the primary tumour, one being atrophic and the other showing necrosis, cyst formation and calcification.

To the 26 cases of clinical regression of untreated renal cancer reported by Everson and Cole (1966) up to the end of 1965 we can add two further cases reported during this period and six others published since then, making a total of 34 examples in the literature up to the end of 1969 (Table VIII). Although unreported cases have undoubtedly been seen, the incidence of spontaneous regression in renal cancer seems to be extraordinarily rare.

In a series of 98 cases of renal cancer presenting with metastases and with a possible follow-up of 12 months reported by Middleton (1967) none showed signs of spontaneous regression with or without nephrectomy: 91 cases were dead by 1 year and all 98 by 2 years. No examples of spontaneous regression were observed by Riches (1963) in 130 cases of renal carcinoma, by Arner and Von Schreeb (1966) in 232 cases, and by Rafla (1970) in 244 cases. One remarkable case of

TABLE VIII.—*Renal Adenocarcinoma*

Reported cases of spontaneous regression of metastases

| Source | Cases |
|---|---|
| Everson and Cole (1966) | 26 |
| Gonick and Jackiw (1964) | 1 |
| Andrews (1965) | 1 |
| Mims *et al.* (1966) | 1 |
| Hudgins and Collins (1966) | 1 |
| Adolfsson (1966) | 2 |
| Markewitz *et al.* (1967) | 1 |
| Robinson (1969) | 1 |
| Total | 34 |

spontaneous regression of skeletal metastases following nephrectomy is reported by Mims *et al.* (1966) in their series of 97 cases of advanced renal cancer of which 57 presented with distant metastases.

In a personal series of almost 200 patients with renal cancer referred for radiotherapy or hormone treatment over the past 10 years, I have seen two cases in whom spontaneous disappearance of pulmonary metastases occurred. In one unimpressive case, a woman of 70, brief spontaneous regression of pulmonary deposits occurred over 3 months and during this time mediastinal nodes increased and vaginal metastases appeared (Bloom, 1967). Events in the second case were far more striking.

*Case F.B., male aged* 55

Nephrectomy for renal carcinoma in presence of bilateral pulmonary metastases. At operation renal tumour involved peritoneum. In keeping with a " wait and see " policy Provera treatment not undertaken at this stage. Two months after operation metastases in left lung smaller; by 5 months all metastases showed signs of regression, and by 7 months all but one had disappeared. Lungs became clear 10 months after nephrectomy. Patient remains in excellent health with clear chest more than 2 years after operation (Bloom and Riddle 1971, not yet published).

In all but two of 36 cases of spontaneous regression (34 in Table VIII plus two mentioned here) improvement was confined to pulmonary metastases (Table IX). Only one example of spontaneous regression of skeletal metastases has been found in the literature (Mims *et al.*, 1966). The other case showing regression of extra-pulmonary deposits, although accepted by Everson and Cole (1966), seems rather dubious; residual or recurrent renal carcinoma tissue was said to have been passed per rectum (Klimpel, 1957).

In the present series of hormone-treated patients clinical evidence of well-marked tumour regression was observed in the lungs (five cases), in large abdominal tumours (six cases), in the kidney itself (one case) and in the skeleton (one case) (Table IX). In a further case not included in this table skeletal deposits remained unchanged for 21 months (case A.D.). It would seem that striking and prolonged regression of widespread metastases involving the brain, chest and skeleton, such as occurred in one of our patients (case D.W.), and the virtual disappearance of a huge abdominal tumour in another (case J.A.D.), have yet to be reported as a spontaneous event in patients with advanced renal cell carcinoma.

The occurrence of spontaneous regression of renal carcinoma, although a rare event, naturally calls for caution in the interpretation of the results of hormone therapy, and emphasises the importance of waiting for clear signs of progressive

TABLE IX.—*Advanced Renal Adenocarcinoma. Site of Regression—Spontaneous and Treated*

| | | Regression | |
|---|---|---|---|
| Series | Cases | Site | Cases |
| Spontaneous | 36 | Lungs | 34 |
| | | Skeleton | 1 |
| | | ? Gut | 1 |
| Hormone-treated | 11 | Lungs | 5 |
| | | Abdomen/Scar | 6 |
| | | Skeleton | 1 |
| | | Brain | 1 |
| | | Primary | 1 |

Some overlap of regression sites in hormone-treated cases.

disease before embarking on such treatment. In view of the rarity of spontaneous improvement of metastatic renal carcinoma it would appear that definite regression in 11 cases, continued tumour standstill for 20 months in two others, and minor or doubtful regressive changes in ten others, in a consecutive series of 80 cases receiving hormone therapy, is more likely to be due to the treatment than to a natural event. This concept is strongly supported by the short interval between commencing or changing hormone treatment and observing clinical or radiological signs of improvement. Furthermore, there are now reports of similar experience from other centres (Melander *et al.*, 1967; Samuels *et al.*, 1968; Talley *et al.*, 1969; Paine *et al.*, 1970).

Although the phenomenon of spontaneous regression of renal cancer has been purposely stressed here, the fact remains that once the presence of visceral or skeletal metastases is established, the disease, in the great majority of untreated patients, advances and causes death within a year or two.

### DISCUSSION AND CONCLUSIONS

The hormonal control of advanced renal cancer, as well as tumours arising in well-established target organs, such as the breast and endometrium, is temporary and largely unpredictable as to frequency and duration. Until a lead can be obtained from further clinical experience or from organ tissue culture and perhaps biochemical studies, the choice of hormone preparation and dose for a particular patient with renal cancer must remain largely empirical. Once it is clear that the disease is advancing and that neither radiotherapy nor surgery are feasible we have started treatment with Provera, 100 mg. thrice daily by mouth. If there is no response to this preparation within 8 weeks, or a shorter time if the patient is rapidly deteriorating, a change has been made to testosterone propionate, 100 mg. intramuscularly on 5 days per week, later reduced to on 3 days per week. If this failed we sometimes tried stilboestrol, 15 to 30 mg. daily, or prednisone using initial doses of 40 mg. daily. Although Samuels *et al.* (1968) remarked on the absence of objective responses in cases of renal cancer treated with Provera by mouth, tumour regression with this progestin can undoubtedly follow oral as well as parenteral treatment.

Because a combination of cortisone and Provera produced a more striking inhibitory effect on the transplantable hamster renal tumour than cortisone alone (Bloom *et al.*, 1963*a*), both hormones were tried together in a few patients who failed to respond to Provera but without success. Significant tumour regression was not observed with corticosteroids or with stilboestrol. Brief subjective improvement was produced occasionally by prednisone.

Although one patient in this series had a remarkable remission lasting 3 years with testosterone (case D.W.), progestins appear to be generally more effective than androgens in the treatment of advanced renal cancer. Only one of 15 cases treated initially with testosterone by Jenkin (1967) showed an objective response.

In two cases of renal carcinoma reported by Talley and his colleagues (1969) objective responses were not apparent until 5 and 8 months respectively had elapsed from onset of hormone therapy. It may therefore be advisable to continue with the initial hormone preparation for longer than the 6 to 8 weeks which has been our policy in the present series. Perhaps the treatment should not be interfered with as long as the patient's general condition remains satisfactory and the metastases stationary.

It must be emphasised that hormone administration is not the treatment of choice for patients with solitary metastases. Such cases are first considered for surgical ablation (*e.g.* lung or brain deposit) or, if this is not feasible, for radiotherapy (*e.g.* spinal or multiple brain deposits). Local irradiation offers a quick and more certain relief of symptoms in metastatic renal cancer than does systemic treatment with hormones or cytotoxic agents.

Prolonged administration of Provera in oral doses of 100 mg. three times daily is well tolerated and does not appear to be associated with serious side-effects. Liver and thyroid function tests and glucose tolerance carried out at intervals in some of our long-term cases remained normal, and there was no fall in plasma cortisol levels nor in 17-hydroxyketo-steroid excretion. Nevertheless, it is advisable to continue to watch for endocrine and perhaps hepatic complications. Macdonald (1970) has reported a reduced adrenal response to metyrapone stimulation tests during treatment with Provera. Stoll *et al.* (1966) have found raised serum transaminase levels together with histological evidence of hepatocellular damage in patients taking the progestin, lynoestrenol, a derivative of 19, nor-testosterone.

We have recently observed degenerative changes in the wall of the ascending aorta with vascular rupture in hamsters treated with large doses of megesterol or melengesterol acetate, compounds with progestational and anti-oestrogenic activity and structurally closely related to Provera (Cobb *et al.*, 1971). Although this complication has fortunately not been observed in any of our patients treated with large doses of Provera, in view of possible changes in the aorta and also the risk of thrombo-embolic disorders related to the progestational component of contraceptive pills (Inman *et al.*, 1970), special attention should be given to the cardiovascular system in advanced cancer cases treated with large doses of progestins that come to autopsy.

Enhanced tumour activity together with marked general deterioration has been seen in two cases in the present series receiving Provera and one testosterone. Bergsjö (1965) has reported tumour acceleration in one of 15 patients with endometrial cancer treated with a progestin. Patients receiving hormones for advanced renal cancer should be watched at frequent intervals during the early stages of treatment for this complication in which event the preparation should be changed (*e.g.* testosterone for Provera, or stilboestrol for testosterone).

Tchao and his colleagues (1968) at this Institute have grown human renal tumour tissue in organ culture. Attempts are being made to use this technique to screen various hormonal preparations against tumour fragments from patients with carcinoma of the kidney by measuring the inhibition of tritiated thymidine incorporated into DNA, and the degree of degeneration seen in ordinary histological sections. In this way it is hoped to find the most effective hormonal agent with which to start treatment in a particular patient. Some of the technical difficultics have now been overcome, but there remains the problem of obtaining a viable piece of tissue with which to attempt *in vitro* growth from a tumour which so often contains necrotic areas.

To date there have been too few tissue culture studies from our cases treated for metastatic renal cell carcinoma with hormonal agents to permit corrclation between *in vitro* and clinical response. More work needs to be done in this interesting field, especially since hormonal agents, at least *in vitro*, appear to act directly on renal tumour cells whilst sparing normal kidney tissue—a potentially most desirable state of affairs for the chemical treatment of cancer.

The inhibitory effect of hormones on renal carcinoma may be a direct one at the cellular level and not mediated through the pituitary. This view is supported by tissue culture studies (Tchao *et al.*, 1968), and also by the absence of signs of pituitary inhibition (based on thyroid function tests, plasma cortisol levels and 17-hydroxycorticosteroid excretion in the urine) in our patients treated with Provera. On the other hand, this compound may possess some pituitary inhibitory effect as shown by gonadal atrophy in animals (Ericsson and Dutt, 1965), a reduced adrenal response to the metyrapone test (Macdonald, 1970) and gonadal suppression in patients with sexual precocity (Kupperman and Epstein, 1962).

Spontaneous regression does not appear to be the explanation for the well-marked objective response seen in 13 of 80 hormone-treated cases of advanced renal cancer in the present series, nor in those reported in the literature. If, indeed, a natural process and not treatment was responsible for the changes observed, then its incidence in renal cell cancer must be far greater than at present generally appreciated. On the contrary, the story of spontaneous regression in renal cancer favours rather than detracts from the concept of hormone-dependency in this disease. The process of spontaneous improvement itsclf in patients with renal cancer appears to be sex-related, 80% of such cases being malcs.

On the basis of the present material and the reports in the literature there seems little doubt that a limited number of renal cell carcinomas can be influenced by hormone therapy, and that this treatment may occasionally offer a new lease of life for a limited period of time to seriously ill patients, even at age 80. Approximately one in five or six patients appear to derive some benefit from hormone treatment. In a few other cases the degree of regression is not significant or clinical improvement is only transitory. Our experience with oral Provera indicates that striking improvement may occur within 4 weeks of starting treatment and, with continued high doses, last for up to 2 years.

Treatment of cancer with progestational agents is an attractive proposition since, unlike cytotoxic agents, they are exceptionally free from side-effects. Patients receiving large doses of Provera, daily over many months or even several years appear to remain in good health.

I am grateful to Dr. R. G. Jacomb and Dr. W. P. Goodyear of Upjohn Ltd. for generous supplies of Provera for experimental and clinical work over the past 10 years. My thanks are due to Dr. I. Hamlin for Fig. 4a and 4b.

### REFERENCES

ADOLFSSON, G.—(1966) *Urol. int.*, **21**, 365.
ANDREWS, J. T.—(1965) *Med. J. Aust.*, **2**, 241.
ARNER, O. AND VON SCHREEB, T.—(1966) *Acta chir. scand.*, **132**, 370.
BABCOCK, J. C., GUTSELL, E. S., HERR, M. E., HOGG, J. A., STUCKI, J. C., BARNES, L. E. AND DULIN, W. E.—(1958) *J. Am. chem. Soc.*, **80**, 2904.
BERGSJÖ, P.—(1965) *Acta endocr., Copenh.*, **49**, 412.
BLOOM, H. J. G.—(1964) in ' Tumours of the Kidney and Ureter '. Edited by E. Riches. London (Livingstone) p. 311.—(1967) in ' Renal Neoplasia '. Edited by J. S. King. Boston (Little, Brown and Co.) p. 605.
BLOOM, H. J. G., BAKER, W. H., DUKES, C. E. AND MITCHLEY, B. C. V.—(1963*b*) *Br. J. Cancer*, **17**, 646.
BLOOM, H. J. G., DUKES, C. E. AND MITCHLEY, B. C. V.—(1963*a*) *Br. J. Cancer*, **17**, 611
BLOOM, H. J. G., ROE, F. J. C. AND MITCHLEY, B. C. V.—(1967) *Cancer, N.Y.*, **20**, 2118.
BLOOM, H. J. G. AND WALLACE, D. M.—(1964) *Br. med. J.*, ii, 476.
COBB, L., BLOOM, H. J. G., ROE, F. J. C. AND MACKENZIE, H. M.—(1971) *Nature, Lond.*, **229**, 50.
ERICSSON, R. J. AND DUTT, R. H.—(1965) *Endocrinology*, **77**, 203.
EVERSON, T. C. AND COLE, W. H.—(1966) ' Spontaneous Regression of Cancer '. Philadelphia (W. B. Saunders Co.) p. 11.
GONICK, P. AND JACKIW, N. M.—(1964) *J. Urol.*, **92**, 270.
HORNING, E.—(1956) *Z. Krebsforsch.*, **61**, 1.
HUDGINS, P. T. AND COLLINS, V. P.—(1966) *Am. J. Roentg.*, **96**, 620.
INMAN, W. H. W., VESSEY, M. P., WESTERHOLM, B. AND ENGELUND, A. (1970) *Br. med. J.*, i, 203.
JENKIN, R. D. T.—(1967) *Br. med. J.*, i, 361.
KIRKMAN, H.—(1959) *Natn. Cancer Inst. Monogr.*, No. 1.
KLIMPEL, K.—(1957) *Z. Urol.*, **50**, 201.
KUPPERMAN, H. S. AND EPSTEIN, J. A.—(1962) *J. clin. Endocr. Metab.*, **22**, 456.
MACDONALD, R. R.—(1970) Paper read at Upjohn Symposium on ' Provera in treatment of some malignancies ', October 27, 1970. Transcript on application to Upjohn, Ltd, Fleming Way, Crawley, Sussex.
MARKEWITZ, M., TAYLOR, D. A. AND VEENEMA, R. J.—(1967) *Cancer, N.Y.*, **20**, 1147.
MATTHEWS, V. S., KIRKMAN, H. AND BACON, R. L.—(1947) *Proc. Soc. exp. Biol. Med.*, **66**, 195.
MELANDER, O., NOTTER, G. AND VON SCHREEB, T.—(1967) *Nord. Med.*, **78**, 1309.
MIDDLETON, R. G.—(1967) in ' Renal Neoplasia '. Edited by J. S. King. Boston (Little, Brown and Co.) p. 483.
MIMS, M. M., CHRISTENSON, B., SCHLUMBERGER, F. C. AND GOODWIN, W. E.—(1966) *J. Urol.*, **95**, 10.
PAINE, C. H., WRIGHT, F. W. AND ELLIS, F.—(1970) *Br. J. Cancer* **24** 277.
RAFLA, S.—(1970) *Cancer, N.Y.*, **25**, 26.
RICHES, E. W.—(1963) *Ann. R. Coll. Surg.*, **32**, 201.
ROBINSON, C. E.—(1969) *Can. med. Ass. J.*, **100**, 297.
ROYCE, R. AND TORMEY, A.—(1955) *J. Urol.*, **74**, 23.
SAMUELS, M. L., SULLIVAN, P. AND HOWE, C. D.—(1968) *Cancer, N.Y.*, **22**, 525.
SHERMAN, A. I.—(1966) *Obstet. Gynec., N.Y.*, **28**, 309.
STOLL, B. A , ANDREWS, J. T. AND MOTTERAM, R.—(1966) *Br. med. J.*, i, 960.
TALLEY, R. W., MOORHEAD, E. L., TUCKER, W. G., SAN DIEGO, E. L. AND BRENNAN, M. J.—(1969) *J. Am. med. Ass.*, **207**, 322.
TCHAO, R., EASTY, G. C., AMBROSE, E. J., RAVEN, R. W. AND BLOOM, H. J. G.—(1968) *Eur. J. Cancer*, **4**, 39.
WOODRUFF, M. W., WAGLE, D., GAILANI, S. D. AND JONES, R.—(1967) *J. Urol.*, **97**, 611.